# Curvature-Dimension Conditions for Symmetric Quantum Markov Semigroups

**DOI:** 10.1007/s00023-022-01220-x

**Published:** 2022-08-08

**Authors:** Melchior Wirth, Haonan Zhang

**Affiliations:** Institute of Science and Technology Austria (ISTA), Am Campus 1, 3400 Klosterneuburg, Austria

**Keywords:** Primary 81S22, Secondary 46L57, 47C99, 49Q22, 81R05

## Abstract

Following up on the recent work on lower Ricci curvature bounds for quantum systems, we introduce two noncommutative versions of curvature-dimension bounds for symmetric quantum Markov semigroups over matrix algebras. Under suitable such curvature-dimension conditions, we prove a family of dimension-dependent functional inequalities, a version of the Bonnet–Myers theorem and concavity of entropy power in the noncommutative setting. We also provide examples satisfying certain curvature-dimension conditions, including Schur multipliers over matrix algebras, Herz–Schur multipliers over group algebras and generalized depolarizing semigroups.

## Introduction

Starting with the celebrated work by Lott–Villani [29] and Sturm [34, 35], recent years have seen a lot of research interest in extending the notion of Ricci curvature, or more precisely lower Ricci curvature bounds, beyond the realm of classical differential geometry to spaces with singularities [2–4, 16], discrete spaces [17, 30, 31] or even settings where there is no underlying space at all as for example in noncommutative geometry [11, 12, 15, 26, 32, 38, 39].

Most of these approaches take as their starting point either the characterization of lower Ricci curvature bound in terms of convexity properties of the entropy on Wasserstein space [37] or in terms of Bakry–Émery’s $\Gamma _2$-criterion [6], which derives from Bochner’s formula, and in many settings, these two approaches yield equivalent or at least closely related notions of lower Ricci curvature bounds.

One of the reasons to seek to extend the notion of Ricci curvature beyond Riemannian manifolds is that lower Ricci curvature bounds have strong geometric consequences and are a powerful tool in proving functional inequalities. This motivated the investigation of lower Ricci curvature bounds in the noncommutative setting, or for quantum Markov semigroups.

From a positive noncommutative lower Ricci curvature bound in terms of the $\Gamma _2$-condition, Junge and Zeng [21, 22] derived a $L_p$-Poincaré-type inequality and transportation inequalities, and under such non-negative lower Ricci curvature bounds Junge and Mei proved $L_p$-boundedness of Riesz transform [20]. Following Lott–Sturm–Villani, Carlen and Maas [10–12] studied the noncommutative lower Ricci curvature bound via the geodesic semi-convexity of entropy by introducing a noncommutative analog of the 2-Wasserstein metric. The similar approach was carried out by the first-named author in the infinite-dimensional setting in [38]. These two notions of lower Ricci curvature bounds are in general different, but they can both be characterized in terms of a gradient estimate [12, 38, 39]. A stronger notion of lower Ricci curvature bound, which implies the bound in terms of $\Gamma _2$-condition and in terms of transportation, was introduced by Li, Junge and LaRacuente [26]. See also the further work of Li [25], and Brannan, Gao and Junge [7, 8].

However, for many applications in geometric consequences such as the Bonnet–Myers theorem, and functional inequalities such as the concavity of entropy power, a lower bound on the Ricci curvature is not sufficient, but one needs an upper bound on the dimension as well. This leads to the curvature-dimension condition, whose noncommutative analog will be the main object of this article. As a finite-dimensional analog of lower Ricci curvature bounds, the curvature-dimension condition also admits various characterizations. Similar to the “infinite-dimensional” setting, two main approaches describing curvature-dimension conditions are $\Gamma _2$-criterion following Bakry–Émery and convexity properties of entropy on the 2-Wasserstein space in the spirit of Lott–Sturm–Villani. For metric measure spaces, the equivalence of various characterizations on curvature-dimension conditions and their applications have been extensively studied beginning with [16].

While the notion of dimension is built into the definition of manifolds, it is not obvious in the extended settings and requires new definitions. The goal of this article is to provide such a definition of dimension (upper bounds) in the context of quantum Markov semigroups in a way that it fits well with the previously developed notions of lower Ricci curvature bounds in this framework. This definition allows us to prove interesting consequences on the geometry of the state space as well as some functional inequalities.

Furthermore, for quantum Markov semigroups satisfying an intertwining condition, which already appeared in [11] and subsequent work, we provide an easily verifiable upper bound on the dimension, namely the number of partial derivatives in the Lindblad form of the generator. This sufficient condition enables us to prove the curvature-dimension condition in various concrete examples such as quantum Markov semigroups of Schur multipliers and semigroups generated by conditionally negative definite length functions on group algebras.

It should be mentioned that a notion of dimension for a quantum diffusion semigroup already appeared implicitly in the work of König and Smith on the quantum entropy power inequality [24]. In particular, from their entropy power inequality one may also derive the concavity of entropy power for the associated quantum diffusion semigroup. See [1, 14, 19] for more related work. This example fits conceptually well with our framework as it satisfies the intertwining condition and the dimension in the entropy power considered there is the number of partial derivatives in the Lindblad form of the generator, although the semigroup acts on an infinite-dimensional algebra and is therefore not covered by our finite-dimensional setting. Here we consider the concavity of the entropy power for arbitrary symmetric quantum Markov semigroups over matrix algebras.

In this paper we will focus on two noncommutative analogues of curvature-dimension conditions: the Bakry–Émery curvature dimension condition BE(*K*, *N*), formulated via the $\Gamma _2$-condition, and the gradient estimate GE(*K*, *N*), which is in the spirit of Lott–Sturm–Villani when the reference operator mean is chosen to be the logarithmic mean. They are generalizations of “infinite-dimensional” notions BE($K,\infty$) and GE($K,\infty$) in previous work, but let us address one difference in the “finite-dimensional” setting, i.e. $N<\infty$. As we mentioned above, in the “infinite-dimensional” case, i.e. $N=\infty$, GE($K,\infty$) recovers BE($K,\infty$) if the operator mean is the left/right trivial mean. However, this is not the case when $N<\infty$; BE(*K*, *N*) is stronger than GE(*K*, *N*) for the left/right trivial mean.

This article is organized as follows. Section 2 collects preliminaries about quantum Markov semigroups and noncommutative differential calculus that are needed for this paper. In Sect. 3 we study the noncommutative Bakry–Émery curvature-dimension condition BE(*K*, *N*), its applications and the complete version. In Sect. 4 we investigate the noncommutative gradient estimate GE(*K*, *N*) for arbitrary operator means, give an equivalent formulation in the spirit of the $\Gamma _2$-criterion, and also introduce their complete form. Section 5 is devoted to the gradient estimate GE(*K*, *N*), its connection to the geodesic (*K*, *N*)-convexity of the (relative) entropy and applications to dimension-dependent functional inequalities. In Sect. 6 we give some examples of quantum Markov semigroups for which our main results apply. In Sect. 7 we discuss how to extend the theory from this article to quantum Markov semigroups that are not necessarily tracially symmetric and explain the main challenge in this case.

## Quantum Markov Semigroups and Noncommutative Differential Calculus

In this section we give some background material on quantum Markov semigroups, their generators, first-order differential calculus and operator means.

### Quantum Markov Semigroups

Throughout this article we fix a finite-dimensional von Neumann algebra $\mathcal {M}$ with a faithful tracial state $\tau$. By the representation theory of finite-dimensional $C^*$-algebras, $\mathcal {M}$ is of the form $\bigoplus _{j=1}^n M_{k_j}(\mathbb {C})$ and $\tau =\bigoplus _{j=1}^n \alpha _j \mathrm {tr}_{M_{k_j}(\mathbb {C})}$ with $\alpha _j\ge 0$, $\sum _{j=1}^n \alpha _j k_j=1$. Here $M_n(\mathbb {C})$ denotes the full *n*-by-*n* matrix algebra and $\mathrm {tr}_{M_{n}(\mathbb {C})}$ is the usual trace over $M_{n}(\mathbb {C})$.

Denote by $\mathcal {M}_+$ the set of positive semi-definite matrices in $\mathcal {M}$. A density matrix is a positive element $\rho \in \mathcal {M}$ with $\tau (\rho )=1$. The set of all density matrices is denoted by $\mathcal {S(M)}$ and the set of all invertible density matrices by $\mathcal {S}_+(\mathcal {M})$. We write $L_2(\mathcal {M},\tau )$ for the Hilbert space obtained by equipping $\mathcal {M}$ with the inner product

$$\begin{aligned} \langle \cdot ,\cdot \rangle :\mathcal {M}\times \mathcal {M}\rightarrow \mathbb {C},\,(x,y)\mapsto \tau (x^*y). \end{aligned}$$

The adjoint of a linear operator $T:\mathcal {M}\rightarrow \mathcal {M}$ with respect to this inner product is denoted by $T^\dagger$. We write $\mathrm {id}$ for the identity operator, with an index indicating on which space it acts if necessary.

A family $(P_t)_{t\ge 0}$ of linear operators on $\mathcal {M}$ is called a *quantum Markov semigroup* if

- $$P_0=\mathrm {id}_\mathcal {M}$$, $$P_{s+t}=P_sP_t$$ for $$s,t\ge 0$$,
- $P_t$ is completely positive for every $t\ge 0$,
- $$P_t {\mathbf {1}}={\mathbf {1}}$$ for every $$t\ge 0$$,
- $$t\mapsto P_t$$ is continuous.

The generator of $(P_t)$ is

$$\begin{aligned} \mathcal {L}:\mathcal {M}\rightarrow \mathcal {M},\,\mathcal {L}x=\lim _{t\searrow 0}\frac{1}{t}(x-P_t(x)). \end{aligned}$$

It is the unique linear operator on $\mathcal {M}$ such that $P_t=e^{-t\mathcal {L}}$. Let us remark that sign conventions differ and sometimes $-\mathcal {L}$ is called the generator of $(P_t)$.

Let $\sigma \in \mathcal {S}_+(\mathcal {M})$. The quantum Markov semigroup $(P_t)$ is said to satisfy the $\sigma$-*detailed balance condition* ($\sigma$-DBC) if

$$\begin{aligned} \tau (P_t(x)y\sigma )=\tau (x P_t(y)\sigma ) \end{aligned}$$

for $x,y\in \mathcal {M}$ and $t\ge 0$. In the special case $\sigma ={\mathbf {1}}$ we say that $(P_t)$ is *tracially symmetric* or *symmetric*, and denote

$$\begin{aligned} \mathcal {E}(a,b):=\langle a,\mathcal {L}b \rangle . \end{aligned}$$

A tracially symmetric quantum Markov semigroup $(P_t)$ is *ergodic* if ${\mathbf {1}}$ is the unique invariant state of $(P_t)$.

Although it is not necessary to *formulate* the curvature-dimension conditions, we will deal exclusively with tracially symmetric quantum Markov semigroups since all examples where we can verify the conditions fall into that class. As a special case of Alicki’s theorem [5, Theorem 3] (see also [11, Theorem 3.1]) the generator $\mathcal {L}$ of a tracially symmetric quantum Markov semigroup on $\mathcal {M}=M_n(\mathbb {C})$ is of the form

$$\begin{aligned} \mathcal {L}=\sum _{j\in \mathcal {J}}\partial _j^\dagger \partial _j, \end{aligned}$$

where $\mathcal {J}$ is a finite index set, $\partial _j=[v_j,\cdot \,]$ for some $v_j\in \mathcal {M}$, and for every $j\in \mathcal {J}$ there exists a unique $j^*\in \mathcal {J}$ such that $v_j^*=v_{j^*}$. We call the operators $\partial _j$ *partial derivatives*. Using the derivation operator $\partial :=(\partial _j)_{j\in \mathcal {J}}:\mathcal {M}\rightarrow {\hat{\mathcal {M}}}:=\oplus _{j\in \mathcal {J}}\mathcal {M}$, we may also write $\mathcal {L}=\partial ^\dagger \partial$.

### Noncommutative Differential Calculus and Operator Means

Let us shortly recall the definition and some basic properties of operator means. Let $\mathcal {H}$ be an infinite-dimensional Hilbert space. A map $\Lambda :B(\mathcal {H})_+\times B(\mathcal {H})_+\rightarrow B(\mathcal {H})_+$ is called an *operator connection* if it satisfies the following properties.

- *monotonicity*: if $A\le C$ and $B\le D$, then $\Lambda (A,B)\le \Lambda (C,D)$,
- *transformer inequality*: $C \Lambda (A,B)C\le \Lambda (C A C,C B C)$ for any $A,B,C\in B(\mathcal {H})_+$,
- *continuity*: $A_n\searrow A$ and $B_n\searrow B$ imply $\Lambda (A_n,B_n)\searrow \Lambda (A,B)$.

An operator connection $\Lambda$ is called an *operator mean* if it additionally satisfies

(d) $$\Lambda (\mathrm {id}_{\mathcal {H}},\mathrm {id}_{\mathcal {H}})=\mathrm {id}_{\mathcal {H}}$$.

Here by $A_n\searrow A$ we mean $A_1\ge A_2\ge \cdots$ and $A_n$ converges strongly to *A*. The operator connection $\Lambda$ is *symmetric* if $\Lambda (A,B)=\Lambda (B,A)$ for all $A,B\in B(\mathcal {H})_+$.

#### Lemma 2.1

Let $\Lambda$ be an operator connection. Then for $\lambda \ge 0$, $A,B,C,D\in B(\mathcal {H})_+$ and unitary $U\in B(\mathcal {H})$, we have

- positive homogeneity: $$\Lambda (\lambda A,\lambda B)=\lambda \Lambda (A,B)$$,
- concavity: $$\Lambda (A,C)+\Lambda (B,D)\le \Lambda (A+B,C+D)$$,
- unitary invariance: $$\Lambda (U^*A U,U^*B U)=U^*\Lambda (A,B)U$$.

If $\Lambda$ is an operator mean, then additionally

(d) $$\Lambda (A,A)=A$$.

#### Proof

See equations ($\hbox {II}_0$), (2.1), Theorem 3.3 and Theorem 3.5 in [23]. $\square$

While operator connections are initially only defined for bounded operators on an infinite-dimensional Hilbert space, one can easily extend this definition to operators on finite-dimensional Hilbert spaces as follows. If $\Lambda$ is an operator connection, $\mathcal {H}$ is a finite-dimensional Hilbert space and $A,B\in B(\mathcal {H})_+$, then one can define $\Lambda (A,B)$ as $V^*\Lambda (VAV^*,VBV^*)V$, where *V* is an isometric embedding of $\mathcal {H}$ into an infinite-dimensional Hilbert space. The unitary invariance from the previous lemma ensures that this definition does not depend on the choice of the embedding *V*.

Let $L(\rho )$ and $R(\rho )$ be the left and right multiplication operators on $L_2(\mathcal {M},\tau )$, respectively, and fix an operator mean $\Lambda$. For $\rho \in \mathcal {M}_+$ we define

$$\begin{aligned} {{\hat{\rho }}}=\Lambda (L(\rho ), R(\rho )). \end{aligned}$$

Of particular interest for us are the cases when $\Lambda$ is the *logarithmic mean*

$$\begin{aligned} \Lambda _{\text {log}}(L(\rho ), R(\rho ))=\int _{0}^{1}L(\rho )^s R(\rho )^{1-s}\,\mathrm{d}s, \end{aligned}$$

or the *left/right trivial mean*

$$\begin{aligned} \Lambda _{\text {left}}(L(\rho ), R(\rho ))=L(\rho ),~~\Lambda _{\text {right}}(L(\rho ), R(\rho ))=R(\rho ). \end{aligned}$$

With $\Lambda =\Lambda _{\text {log}}$ being the logarithmic mean, we have the chain rule identity for $\log$ (see [11, Lemma 5.5] for a proof):

$$\begin{aligned} \partial \rho ={\hat{\rho }}\partial \log \rho =\int _{0}^1\rho ^s(\partial \log \rho ) \rho ^{1-s}\mathrm{d}s. \end{aligned}$$

Here and in what follows, we use the notation

$$\begin{aligned} {\hat{\rho }}(x_1,\dots ,x_n):=({\hat{\rho }} x_1,\dots , {\hat{\rho }}x_n). \end{aligned}$$

## Bakry–Émery Curvature-Dimension Condition BE(*K*, *N*)

This section is devoted to the noncommutative analog of the Bakry–Émery curvature-dimension condition BE(*K*, *N*) defined by the $\Gamma _2$-criterion. After giving the definition, we will show that it is satisfied for certain generators in Lindblad form, where the dimension parameter *N* is given by the number of partial derivatives. We will then prove that $\mathrm {BE}(K,N)$ implies an improved Poincaré inequality. In the final part of this section we study a complete version of $\mathrm {BE}(K,N)$, called $\mathrm {CBE}(K,N)$, and show that it has the expected tensorization properties.

### Bakry–Émery Curvature-Dimension Condition BE(*K*, *N*)

Let $(P_t)$ be a quantum Markov semigroup on $\mathcal {M}$ with generator $\mathcal {L}$. The associated *carré du champ operator* $\Gamma$ is defined as

$$\begin{aligned} \Gamma (a,b):=\frac{1}{2}\left( a^*\mathcal {L}b+(\mathcal {L}a)^*b-\mathcal {L}(a^*b)\right) , \end{aligned}$$

and the *iterated carré du champ operator* $\Gamma _2$ is defined as

$$\begin{aligned} \Gamma _2(a,b):=\frac{1}{2}\left( \Gamma (a,\mathcal {L}b) +\Gamma (\mathcal {L}a,b)-\mathcal {L}\Gamma (a, b)\right) . \end{aligned}$$

As usual, we write $\Gamma (a)$ for $\Gamma (a,a)$ and $\Gamma _2(a)$ for $\Gamma _2(a,a)$.

#### Proposition 3.1

Let $K\in \mathbb {R}$ and $N\in (0,\infty ]$. For a quantum Markov semigroup $(P_t)$ over $\mathcal {M}$ with generator $\mathcal {L}$, the following are equivalent:

- for any $t\ge 0$ and any $a\in \mathcal {M}$:
  $$\begin{aligned} \Gamma (P_t a)\le e^{-2Kt}P_t\Gamma (a)-\frac{1-e^{-2Kt}}{KN}|\mathcal {L}P_t a|^2, \end{aligned}$$
- for any $$a\in \mathcal {M}$$:
  $$\begin{aligned} \Gamma _2(a)\ge K\Gamma (a)+\frac{1}{N}|\mathcal {L}a|^2. \end{aligned}$$

If this is the case, we say the semigroup $(P_t)$ satisfies Bakry–Émery curvature-dimension condition $\mathrm {BE}(K,N)$.

#### Proof

The proof is essentially based on the following identities: For $s\in [0,t]$,

$$\begin{aligned} \frac{\mathrm{d}}{\mathrm{d}s}P_s((P_{t-s}a)^*(P_{t-s}a))=2P_s \Gamma (P_{t-s}a), \end{aligned}$$

and

$$\begin{aligned} \frac{\mathrm{d}}{\mathrm{d}s}P_s \Gamma (P_{t-s}a)=2P_s \Gamma _2(P_{t-s}a), \end{aligned}$$

which follow by direct computations. To prove $(a)\implies (b)$, we set

$$\begin{aligned} \phi (t):=e^{-2Kt}P_t\Gamma (a)-\Gamma (P_t a)-\frac{1-e^{-2Kt}}{KN}|\mathcal {L}P_t a|^2. \end{aligned}$$

Since $\phi (t)\ge 0$ for all $t\ge 0$ and $\phi (0)=0$, we have $\phi '(0)\ge 0$, which is nothing but (b).

To show $(b)\implies (a)$, we put for any $t>0$:

$$\begin{aligned} \qquad \varphi (s):=e^{-2Ks}P_s\Gamma (P_{t-s}a),~~s\in [0,t]. \end{aligned}$$

By the Kadison–Schwarz inequality, $\Phi (b)^*\Phi (b)\le \Phi (b^*b)$ for any unital completely positive map $\Phi$ on $\mathcal {M}$ and $b\in \mathcal {M}$. If we apply this to $P_s$ and use the assumption from (b), we get

$$\begin{aligned} \varphi '(s)= & {} 2e^{-2Ks}P_s\left( \Gamma _2(P_{t-s}a)-K\Gamma (P_{t-s}a)\right) \ge \frac{2e^{-2Ks}}{N}P_s\left( |\mathcal {L}P_{t-s}a|^2\right) \\\ge & {} \frac{2e^{-2Ks}}{N}|\mathcal {L}P_{t}a|^2. \end{aligned}$$

So

$$\begin{aligned} \varphi (t)-\varphi (0)=\int _{0}^{t}\varphi '(s)\mathrm{d}s \ge \frac{2}{N}\int _{0}^{t}e^{-2Ks}\mathrm{d}s|\mathcal {L}P_{t}a|^2 =\frac{1-e^{-2Kt}}{KN}|\mathcal {L}P_{t}a|^2, \end{aligned}$$

which proves (a). $$\square $$

#### Remark 3.2

From the proof one can see that the function

$$\begin{aligned} t\mapsto \frac{1-e^{-2Kt}}{KN}, \end{aligned}$$

in (a) can be replaced by any *f* such that $f(0)=0$ and $f'(0)=2/N$.

#### Remark 3.3

The notion $\mathrm {BE}(K,N)$ is clearly consistent: If $(P_t)$ satisfies $\mathrm {BE}(K,N)$, then it also satisfies $\mathrm {BE}(K',N')$ for all $K'\le K$ and $N'\ge N$.

#### Remark 3.4

While all our examples of quantum Markov semigroups satisfying $\mathrm {BE}$ are tracially symmetric, let us point out that this is not necessary for the definition nor for the results in the rest of this section with the exception of Proposition 3.7. See also the discussion in Sect. 7.

We shall give a sufficient condition for Bakry–Émery curvature-dimension condition BE(*K*, *N*). Before that we need a simple inequality.

#### Lemma 3.5

For any $a_{j},1\le j\le d$, in a C*-algebra, we have

$$\begin{aligned} \sum _{j=1}^{d}|a_{j}|^2\ge \frac{1}{d}\left| \sum _{j=1}^{d}a_{j}\right| ^2. \end{aligned}$$

#### Proof

In fact,

$$\begin{aligned}&d\sum _{j=1}^{d}|a_{j}|^2-\left| \sum _{j=1}^{d}a_{j}\right| ^2 =\frac{1}{2}\sum _{j,k=1}^{d}\left( a_j^*a_j+a_k^*a_k-a_j^*a_k-a_k^*a_j\right) \\&\quad \quad \quad =\frac{1}{2}\sum _{j,k=1}^{d}|a_j-a_k|^2 \ge 0.\square \end{aligned}$$

#### Definition 3.6

Suppose that $\mathcal {L}$ is the generator of the tracially symmetric quantum Markov semigroup $(P_t)$ with the Lindblad form: 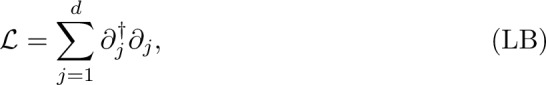 where $\partial _j(\cdot )=[v_j,\cdot ]$ with the adjoint being $\partial _j^\dagger (\cdot )=[v_j^*,\cdot ]$, and $\{v_j\}=\{v_j^*\}$. Then we say $(P_t)$ satisfies the *K*-intertwining condition for some $K\in \mathbb {R}$ if

$$\begin{aligned} \partial _j P_t=e^{-K t}P_t\partial _j,\quad ~~1\le j\le d, \end{aligned}$$

or equivalently

$$\begin{aligned} \partial _j \mathcal {L}=\mathcal {L}\partial _j+K\partial _j,\quad ~~1\le j\le d. \end{aligned}$$

#### Proposition 3.7

Suppose that the generator $\mathcal {L}$ of the tracially symmetric quantum Markov semigroup $(P_t)$ admits the Lindblad form (LB). Then for any *a*,

3.1 $$\begin{aligned} \Gamma _2(a)={\text {Re}}\sum _{j=1}^{d}(\partial _j\mathcal {L}a-\mathcal {L}\partial _j a)^*\partial _j a+\sum _{j,k=1}^{d}|\partial _k^\dagger \partial _j a|^2. \end{aligned}$$

If $(P_t)$ satisfies the *K*-intertwining condition for $K\in \mathbb {R}$, then $(P_t)$ satisfies $\mathrm {BE}(K,d)$.

#### Proof

Note that

$$\begin{aligned} (\partial _j a)^*=a^*v_j^*-v_j^*a^*=-\partial _j^\dagger (a^*). \end{aligned}$$

This, together with the Leibniz rule for $\partial _j$’s (so also $\partial _j^\dagger$’s), and the fact that $\{\partial _j\}=\{\partial _j^\dagger \}$, yields

$$\begin{aligned} \mathcal {L}(a^*b)&=\sum _{j=1}^{d}(\partial _j^\dagger \partial _ja^*)b+a^*\partial _j^\dagger \partial _j b+(\partial _j^\dagger a^*)(\partial _j b)+(\partial _j a^*)(\partial _j^\dagger b)\\&=(\mathcal {L}a)^*b+a^*\mathcal {L}b+\sum _{j=1}^{d}(\partial _j^\dagger a^*)(\partial _j b)+(\partial _j a^*)(\partial _j^\dagger b)\\&=(\mathcal {L}a)^*b+a^*\mathcal {L}b-\sum _{j=1}^{d}\left( (\partial _j a)^*(\partial _j b)+(\partial _j^\dagger a)^*(\partial _j^\dagger b)\right) \\&=(\mathcal {L}a)^*b+a^*\mathcal {L}b-2\sum _{j=1}^{d}(\partial _j a)^*(\partial _j b). \end{aligned}$$

So by definition, the carré du champ operator is given by:

3.2 $$\begin{aligned} \Gamma (a,b) =\frac{1}{2}\left( a^*\mathcal {L}b+(\mathcal {L}a)^*b-\mathcal {L}(a^*b) \right) =\sum _{j=1}^{d}(\partial _j a)^*\partial _j b. \end{aligned}$$

The above computations yield

$$\begin{aligned} \Gamma (a,\mathcal {L}(a))+\Gamma (\mathcal {L}(a),a) =\sum _{j=1}^{d}(\partial _j \mathcal {L}a)^*\partial _j a+(\partial _j a)^*\partial _j \mathcal {L}a =2{\text {Re}}\sum _{j=1}^{d}(\partial _j \mathcal {L}a)^*\partial _j a, \end{aligned}$$

and

$$\begin{aligned} \mathcal {L}\Gamma (a)&=\sum _{j=1}^{d}\mathcal {L}\left( (\partial _j a)^*\partial _j a\right) \\&=\sum _{j=1}^{d}\left( (\mathcal {L}\partial _j a)^*\partial _j a+(\partial _j a)^*\mathcal {L}\partial _j a-2\sum _{k=1}^{d}(\partial _k\partial _j a)^*\partial _k\partial _j a\right) \\&=2{\text {Re}}\sum _{j=1}^{d}(\mathcal {L}\partial _j a)^*\partial _j a -2\sum _{j,k=1}^{d}|\partial _k\partial _j a|^2. \end{aligned}$$

Thus

$$\begin{aligned} \Gamma _2(a) =&\frac{1}{2}\left( \Gamma (a,\mathcal {L}(a))+\Gamma (\mathcal {L}(a),a)-\mathcal {L}\Gamma (a)\right) \\ =&{\text {Re}}\sum _{j=1}^{d}(\partial _j \mathcal {L}a)^*\partial _j a-{\text {Re}}\sum _{j=1}^{d}(\mathcal {L}\partial _j a)^*\partial _j a+\sum _{j,k=1}^{d}|\partial _k\partial _j a|^2\\ =&{\text {Re}}\sum _{j=1}^{d}(\partial _j \mathcal {L}a-\mathcal {L}\partial _j a)^*\partial _j a+\sum _{j,k=1}^{d}|\partial _k^\dagger \partial _j a|^2, \end{aligned}$$

where in the last equality we used again the fact that $\{\partial _j\}=\{\partial _j^\dagger \}$. This proves (3.1). If $(P_t)$ satisfies the *K*-intertwining condition, then

$$\begin{aligned} {\text {Re}}\sum _{j=1}^d(\partial _j \mathcal {L}a-\mathcal {L}\partial _j a)^*(\partial _j a)=K\sum _{j=1}^d (\partial _j a)^*(\partial _j a)=K\Gamma (a). \end{aligned}$$

Moreover, by Lemma 3.5 we get

$$\begin{aligned} \sum _{j,k=1}^d |\partial _k^\dagger \partial _j a|^2 \ge \sum _{j=1}^d |\partial _j^\dagger \partial _j a|^2 \ge \frac{1}{d} \left|\sum _{j=1}^d \partial _j^\dagger \partial _j a\right|^2 =\frac{1}{d} |\mathcal {L}a|^2. \end{aligned}$$

Therefore $$(P_t)$$ satisfies $$\mathrm {BE}(K,d)$$:

$$\begin{aligned} \Gamma _2(a)={\text {Re}}\sum _{j=1}^{d}(\partial _j \mathcal {L}a-\mathcal {L}\partial _j a)^*\partial _j a+\sum _{j,k=1}^{d}|\partial _k^\dagger \partial _j a|^2 \ge K\Gamma (a)+\frac{1}{d} |\mathcal {L}a|^2. \square \end{aligned}$$

### Applications

In this subsection we present two applications of the Bakry–Émery curvature-dimension condition, namely a Poincaré inequality and a Bonnet–Myers theorem.

It is well-known that when $K>0$, the dimensionless bound $\mathrm {BE}(K,\infty )$ implies that the smallest non-zero eigenvalue of the generator is at least *K*. As a simple application of the dimensional variant we show that this bound can be improved.

#### Proposition 3.8

(Poincaré inequality) Let $K>0$ and $N>1$. If $(P_t)$ satisfies $\mathrm {BE}(K,N)$ and $\lambda _1$ is the smallest non-zero eigenvalue of $\mathcal {L}$, then

$$\begin{aligned} \lambda _1\ge \frac{KN}{N-1}. \end{aligned}$$

#### Proof

By $\mathrm {BE}(K,N)$ we have

$$\begin{aligned} \Vert \mathcal {L} a\Vert _2^2=\tau (\Gamma _2(a))\ge K\tau (\Gamma (a))+\frac{1}{N} \tau (|\mathcal {L}a|^2)=K\langle \mathcal {L} a,a\rangle +\frac{1}{N} \Vert \mathcal {L}a\Vert _2^2. \end{aligned}$$

In particular, if $\mathcal {L}a=\lambda _1 a$ and $\Vert a\Vert _2=1$, then

$$\begin{aligned} \lambda _1^2\ge K\lambda _1+\frac{1}{N} \lambda _1^2, \end{aligned}$$

from which the desired inequality follows. $\square$

To state the Bonnet–Myers theorem, we recall the definition of the metric $d_\Gamma$ on the space of density matrices that is variously known as *quantum* $L^1$-*Wasserstein distance*, *Connes distance* or *spectral distance*. It is given by

$$\begin{aligned} d_\Gamma (\rho _0,\rho _1)=\sup \{\tau (a(\rho _1-\rho _0))\mid a=a^*\in \mathcal {M},\,\Gamma (a)\le {\mathbf {1}}\} \end{aligned}$$

for $$\rho _0,\rho _1\in \mathcal {S(M)}$$.

#### Proposition 3.9

Let $K,N\in (0,\infty )$. If a symmetric quantum Markov semigroup $(P_t)$ is ergodic and satisfies Bakry–Émery curvature-dimension condition $\mathrm {BE}(K,N)$, then

$$\begin{aligned} d_\Gamma (\rho ,{\mathbf {1}})\le \frac{\pi }{2}\sqrt{\frac{N}{K}} \end{aligned}$$

for all $$\rho \in \mathcal {S}(\mathcal {M})$$.

In particular,

$$\begin{aligned} \sup _{\rho _0,\rho _1\in \mathcal {S(M)}}d_\Gamma (\rho _0,\rho _1)\le \pi \sqrt{\frac{N}{K}}. \end{aligned}$$

#### Proof

The proof follows the same line as that of [28, Theorem 2.4]. The condition $\mathrm {BE}(K,N)$ implies

$$\begin{aligned} \frac{1-e^{-2Kt}}{KN}(\mathcal {L}P_t a)^2\le e^{-2Kt}P_t\Gamma (a), \end{aligned}$$

for any $a=a^*\in \mathcal {M}$. If $\Gamma (a)\le {\mathbf {1}}$, we have

$$\begin{aligned} \Vert \mathcal {L}P_t a\Vert \le \sqrt{KN}\sqrt{\frac{1}{e^{2Kt}-1}}. \end{aligned}$$

Thus for any $\rho \in \mathcal {S}(\mathcal {M})$,

$$\begin{aligned} |\tau ((P_t a-a)\rho )|\le \int _0^t \left|\frac{\mathrm{d}}{\mathrm{d}s}\tau ((P_s a)\rho )\right|\,\mathrm{d}s\le \sqrt{KN}\int _0^\infty \frac{1}{\sqrt{e^{2Ks}-1}}\,\mathrm{d}s=\frac{\pi }{2}\sqrt{\frac{N}{K}}. \end{aligned}$$

Therefore

$$\begin{aligned} \tau (a(\rho -{\mathbf {1}}))=\tau ((a-P_t a)\rho )+\tau (P_t a(\rho -{\mathbf {1}})) \le \frac{\pi }{2}\sqrt{\frac{N}{K}}+\tau (a(P_t\rho -{\mathbf {1}})). \end{aligned}$$

Since $(P_t)$ is assumed to be ergodic, we have $P_t\rho \rightarrow {\mathbf {1}}$ as $t\rightarrow \infty$, and we end up with

$$\begin{aligned} d_\Gamma (\rho ,{\mathbf {1}})= \sup _{\Gamma (a)\le 1}\tau (a(\rho -{\mathbf {1}}))\le \frac{\pi }{2}\sqrt{\frac{N}{K}}. \end{aligned}$$

$$\square $$

#### Remark 3.10

If $(P_t)$ is not ergodic, then the same argument gives

$$\begin{aligned} d_\Gamma (\rho ,E(\rho ))\le \frac{\pi }{2}\sqrt{\frac{N}{K}}, \end{aligned}$$

where $E(\rho )=\lim _{t\rightarrow \infty }P_t(\rho )$. In particular,

$$\begin{aligned} d_\Gamma (\rho _0,\rho _1)\le \pi \sqrt{\frac{N}{K}} \end{aligned}$$

whenever $$E(\rho _0)=E(\rho _1)$$.

### Complete BE(*K*, *N*)

In many applications it is desirable to have estimates that are tensor-stable in the sense that they hold not only for $(P_t)$, but also for $(P_t\otimes \mathrm {id}_{M_n(\mathbb {C})})$ with a constant independent of $n\in \mathbb {N}$, as this allows to analyze complex composite systems by studying their subsystems separately.

Even in the case $N=\infty$, it seems to be unknown if this tensor stability holds for the Bakry–Émery estimate. For that reason we introduce the complete Bakry–Émery estimate $\mathrm {CBE}(K,N)$, which has this tensor stability by definition. We will show that this stronger estimate also holds for quantum Markov semigroups satisfying the *K*-intertwining condition, and moreover, this estimate behaves as expected under arbitrary tensor products.

#### Definition 3.11

Let $K\in \mathbb {R}$ and $N> 0$. We say that the quantum Markov semigroup $(P_t)$ satisfies $\mathrm {CBE}(K,N)$ if

$$\begin{aligned}{}[\Gamma (P_t x_j,P_t x_k)]_{j,k}\le e^{-2Kt}[P_t \Gamma (x_j,x_k)]_{j,k}-\frac{1-e^{-2Kt}}{KN} [(\mathcal {L}P_t x_j)^*(\mathcal {L}P_t x_k)]_{j,k}, \end{aligned}$$

for all $x_1,\dots ,x_n\in \mathcal {M}$ and $t> 0$.

Just as in Proposition 3.1 one can show that $\mathrm {CBE}(K,N)$ is equivalent to

$$\begin{aligned} {[}\Gamma _2(x_j,x_k)]_{j,k}\ge K[\Gamma (x_j,x_k)]_{j,k}+\frac{1}{N} [(\mathcal {L}x_j)^*(\mathcal {L}x_k)]_{j,k} \end{aligned}$$

for all $x_1,\dots ,x_n\in \mathcal {M}$ and $t\ge 0$.

For $N=\infty$, this criterion was introduced in [21] for group von Neumann algebras under the name *algebraic* $\Gamma _2$-*condition*.

To show that $\mathrm {CBE}(K,N)$ for $(P_t)$ is equivalent to $\mathrm {BE}(K,N)$ for $(P_t\otimes \mathrm {id}_{M_n(\mathbb {C})})$ with constants independent of *n*, we need the following elementary lemma.

#### Lemma 3.12

Let $\mathcal {A},\mathcal {B}$ be two C*-algebras. If $x=[x_{jk}]\in M_n(\mathcal {A})$, $y=[y_{jk}]\in M_n(\mathcal {B})$ are positive, then

$$\begin{aligned} \sum _{j,k}x_{jk}\otimes y_{jk}\ge 0. \end{aligned}$$

#### Proof

By assumption there are $a=[a_{jk}]\in M_n(\mathcal {A})$, $b=[b_{jk}]\in M_n(\mathcal {B})$ such that

$$\begin{aligned} x_{jk}&=\sum _{l}a_{lj}^*a_{lk},\\ y_{jk}&=\sum _{m}b_{mj}^*b_{mk}. \end{aligned}$$

Thus

$$\begin{aligned} \sum _{j,k}x_{jk}\otimes y_{jk}&=\sum _{j,k,l,m}a_{lj}^*a_{lk}\otimes b_{mj}^*b_{mk}\\&=\sum _{l,m}\left( \sum _j a_{lj}^*\otimes b_{mj}^*\right) \left( \sum _k a_{lk}\otimes b_{mk}\right) \\&=\sum _{l,m}\left|\sum _j a_{lj}\otimes b_{mj}\right|^2\\&\ge 0. \end{aligned}$$

$$\square $$

#### Proposition 3.13

Let $(P_t)$ be a quantum Markov semigroup on $\mathcal {M}$. For $K\in \mathbb {R}$ and $N\in (0,\infty ]$, the following assertions are equivalent:

- $$(P_t)$$ satisfies $$\mathrm {CBE}(K,N)$$.
- $(P_t\otimes \mathrm {id}_{M_n(\mathbb {C})})$ satisfies $\mathrm {BE}(K,N)$ for all $n\in \mathbb {N}$.

#### Proof

(a)$\implies$(b): Write $\Gamma ,\Gamma _2$ for the (iterated) carré du champ associated with $(P_t)$ and $\Gamma ^\otimes ,\Gamma _2^\otimes$ for the same forms associated with $(P_t\otimes \mathrm {id}_{M_n(\mathbb {C})})$.

A direct computation shows

$$\begin{aligned}&\Gamma _2^\otimes \left( \sum _j x_j\otimes y_j\right) =\sum _{j,k}\Gamma _2(x_j,x_k)\otimes y_j^*y_k,\\&\Gamma ^\otimes \left( \sum _j x_j\otimes y_j\right) =\sum _{j,k}\Gamma (x_j,x_k)\otimes y_j^*y_k,\\&\qquad \left|(\mathcal {L}\otimes \mathrm {id}_{M_n(\mathbb {C})})\left( \sum _j x_j\otimes y_j\right) \right|^2=\sum _{j,k}(\mathcal {L}x_j)^*(\mathcal {L}x_k)\otimes y_j^*y_k. \end{aligned}$$

Hence

$$\begin{aligned}&\Gamma _2^\otimes \left( \sum _j x_j\otimes y_j\right) -K\Gamma ^\otimes \left( \sum _j x_j\otimes y_j\right) \\&\qquad -\frac{1}{N} \left|(\mathcal {L}\otimes \mathrm {id}_\mathcal {N})\left( \sum _j x_j\otimes y_j\right) \right|^2\\&\quad =\sum _{j,k}\left( \Gamma _2(x_j,x_k)-K\Gamma (x_j,x_k)-\frac{1}{N}(\mathcal {L}x_j)^*(\mathcal {L}x_k)\right) \otimes y_j^*y_k, \end{aligned}$$

and the result follows from Lemma 3.12 and (a).

(b)$\implies$(a): Let $x=\sum _j x_j\otimes |1\rangle \langle j|$. The computations from (a)$\implies$(b) show

$$\begin{aligned} \Gamma _2^\otimes (x)=\sum _{j,k}\Gamma _2(x_j,x_k)\otimes |j\rangle \langle k| \end{aligned}$$

and similar formulas for $\Gamma ^\otimes$ and $\mathcal {L}\otimes \mathrm {id}_{M_n(\mathbb {C})}$. Using the $*$-isomorphism $\mathcal {M}\otimes M_n(\mathbb {C})\rightarrow M_n(\mathcal {M}),\,\sum _{j,k}x_{jk}\otimes |j\rangle \langle k|\mapsto [x_{jk}]_{j,k}$, assertion (a) follows. $\square$

In the following two results we will give two classes of examples for which the condition $\mathrm {CBE}$ is satisfied.

#### Proposition 3.14

Suppose that the generator $\mathcal {L}$ of the quantum Markov semigroup $(P_t)$ admits the Lindblad form (LB) with *d* partial derivatives $\partial _1,\dots ,\partial _d$. If $(P_t)$ satisfies the *K*-intertwining condition for $K\in \mathbb {R}$, then $(P_t)$ satisfies $\mathrm {CBE}(K,d)$.

#### Proof

A direct computation shows that $\mathcal {L}\otimes \mathrm {id}_{M_n(\mathbb {C})}$ admits a Lindblad form with partial derivatives $\partial _1\otimes \mathrm {id}_{M_n(\mathbb {C})},\dots ,\partial _d\otimes \mathrm {id}_{M_n(\mathbb {C})}$. Now the claim is a direct consequence of Propositions 3.7 and 3.13. $\square$

#### Proposition 3.15

If $\mathcal {M}$ is commutative and $(P_t)$ satisfies $\mathrm {BE}(K,N)$, then it also satisfies $\mathrm {CBE}(K,N)$.

#### Proof

By assumption, $\mathcal {M}\cong C(X)$ for a compact space *X*. We have to show

$$\begin{aligned} {[}\Gamma _2(f_j,f_k)(x)]_{j,k}\ge K[\Gamma (f_j,f_k)(x)]_{j,k}+\frac{1}{N} [\overline{(\mathcal {L}f_j)(x)} (\mathcal {L}f_k)(x)]_{j,k} \end{aligned}$$

for $x\in X$, which follows from

$$\begin{aligned}&\sum _{j,k}\overline{\alpha _j}\alpha _k \Gamma _2(f_j,f_k)(x)\\&\quad =\Gamma _2\left( \sum _j \alpha _j f_j\right) (x)\\&\quad \ge K\Gamma \left( \sum _j \alpha _j f_j\right) (x)+\frac{1}{N} \left|\mathcal {L}\left( \sum _j \alpha _j f_j\right) (x)\right|^2\\&\quad =K\sum _{j,k}\overline{\alpha _j}\alpha _k \Gamma (f_j,f_k)(x)+\frac{1}{N} \sum _{j,k}\overline{\alpha _j}\alpha _k \overline{(\mathcal {L}f_j)(x)}(\mathcal {L}f_k)(x) \end{aligned}$$

for any $$\alpha _j\in \mathbb {C}$$. $$\square $$

Before we state the tensorization property of $\mathrm {CBE}$, we need another elementary inequality.

#### Lemma 3.16

Let $\mathcal {A}$ be a C*-algebra. If $a,b\in \mathcal {A}$ and $\lambda >0$, then

$$\begin{aligned} |a+b|^2\le (1+\lambda )|a|^2+(1+\lambda ^{-1})|b|^2. \end{aligned}$$

#### Proof

In fact,

$$\begin{aligned}&(1+\lambda )|a|^2+(1+\lambda ^{-1})|b|^2\\&\quad =|a+b|^2+\lambda |a|^2+\lambda ^{-1}|b|^2-a^*b-b^*a\\&\quad =|a+b|^2+|\lambda ^{1/2}a-\lambda ^{-1/2}b|^2\\&\quad \ge |a+b|^2. \end{aligned}$$

$$\square $$

#### Proposition 3.17

Let $\mathcal {M}$, $\mathcal {N}$ be finite-dimensional von Neumann algebras and let $(P_t)$, $(Q_t)$ be tracially symmetric quantum Markov semigroups on $\mathcal {M}$ and $\mathcal {N}$, respectively. If $(P_t)$ satisfies $\mathrm {CBE}(K,N)$ and $(Q_t)$ satisfies $\mathrm {CBE}(K',N')$, then $(P_t\otimes Q_t)$ satisfies $\mathrm {CBE}(\min \{K,K'\},N+N')$.

#### Proof

We use superscripts for the (iterated) carré du champ to indicate the associated quantum Markov semigroup. Let $\kappa =\min \{K,K'\}$. We have

$$\begin{aligned} \Gamma _2^{P\otimes Q}-\kappa \Gamma ^{P\otimes Q} =(\Gamma _2^{P\otimes \mathrm {id}_\mathcal {N}}-\kappa \Gamma ^{P\otimes \mathrm {id}_\mathcal {N}})+(\Gamma _2^{\mathrm {id}_\mathcal {M}\otimes Q}-\kappa \Gamma ^{\mathrm {id}_\mathcal {M}\otimes Q})+2\Gamma ^P\otimes \Gamma ^Q, \end{aligned}$$

where

$$\begin{aligned} (\Gamma ^P\otimes \Gamma ^Q)\left( \sum _j x_j\otimes y_j\right) :=\sum _{j,k}\Gamma ^P(x_j,x_k)\otimes \Gamma ^Q(y_j,y_k). \end{aligned}$$

By $\mathrm {CBE}(\kappa ,N)$ for $(P_t)$ and $\mathrm {CBE}(\kappa ,N')$ for $(Q_t)$ we have

$$\begin{aligned}&(\Gamma _2^{P\otimes \mathrm {id}_\mathcal {N}}-\kappa \Gamma ^{P\otimes \mathrm {id}_\mathcal {N}})\left( \sum _j x_j\otimes y_j\right) \ge \frac{1}{N}\left|\sum _j \mathcal {L}_Px_j\otimes y_j\right|^2,\\&\quad (\Gamma _2^{\mathrm {id}_\mathcal {M}\otimes Q}-\kappa \Gamma ^{\mathrm {id}_\mathcal {M}\otimes Q})\left( \sum _j x_j\otimes y_j\right) \ge \frac{1}{N'}\left|\sum _j x_j\otimes \mathcal {L}_Q y_j\right|^2. \end{aligned}$$

Moreover,

$$\begin{aligned} (\Gamma ^P\otimes \Gamma ^Q)\left( \sum _j x_j\otimes y_j\right) \ge 0 \end{aligned}$$

by Lemma 3.12.

Finally,

$$\begin{aligned}&\frac{1}{N}\left|\sum _j \mathcal {L}_Px_j\otimes y_j\right|^2+\frac{1}{N'}\left|\sum _j x_j\otimes \mathcal {L}_Q y_j\right|^2\\&\quad \ge \frac{1}{N+N'}\left|\sum _j \mathcal {L}_P x_j\otimes y_j+x_j\otimes \mathcal {L}_Q y_j\right|^2 \end{aligned}$$

by Lemma 3.16, which shows $\mathrm {BE}(\kappa ,N+N')$ for $(P_t\otimes Q_t)$. To prove $\mathrm {CBE}(\kappa ,N+N')$, we can simply apply the same argument to $(P_t\otimes \mathrm {id}_{M_n(\mathbb {C})})$ and $(Q_t\otimes \mathrm {id}_{M_n(\mathbb {C})})$ for arbitrary $n\in \mathbb {N}$. $\square$

## The Gradient Estimate $\mathrm {GE}(K,N)$

### Gradient Estimate $\mathrm {GE}(K,N)$ and a Sufficient Condition

In [10–12, 38], a noncommutative analog of the 2-Wasserstein metric was constructed on the set of quantum states. Among other things, it gives rise to a notion of (entropic) lower Ricci curvature bound via geodesic semi-convexity of the entropy. This allows to prove a number of functional inequalities under strictly positive lower Ricci curvature bound, including the modified log-Sobolev inequality that (seemingly) cannot be produced under the Bakry–Émery curvature-dimension condition BE($K,\infty$).

This entropic lower Ricci curvature bound is captured in the following gradient estimate 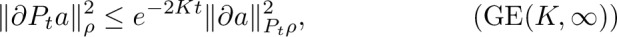 or equivalently

4.1 $$\begin{aligned} {\text {Re}}\langle \partial \mathcal {L}a,{\hat{\rho }}\partial a\rangle +\frac{1}{2}\left\langle \frac{\mathrm{d}}{\mathrm{d}t}\big |_{t=0}\widehat{P_t \rho }\partial a,\partial a\right\rangle \ge K \Vert \partial a\Vert _{\rho }^2, \end{aligned}$$

where the notations ${\hat{\rho }}$ and $\Vert \cdot \Vert _{\rho }$ correspond to the logarithmic mean $\Lambda _{\text {log}}$. Recall Sect. 2 for more details. The fact that logarithmic mean comes into play lies in the use of chain rule

$$\begin{aligned} {\hat{\rho }}\partial _j \log \rho =\partial _j\rho ,~~1\le j\le d. \end{aligned}$$

In fact, for the gradient estimate (GE$(K,\infty )$) and its equivalent form 4.1 one can work with any operator mean. This not only makes the theory more flexible, but also includes the Bakry–Émery curvature-dimension condition BE($K,\infty$) as a special case. Indeed, one recovers BE($K,\infty$) by replacing the logarithmic mean in (4.1) with the left/right trivial mean. In the next section we discuss the connection between GE(*K*, *N*) and (*K*, *N*)-convexity of the (relative) entropy.

The study of (GE$(K,\infty )$) for arbitrary operator means was started in [38, 39]. Here we continue to work within this framework and focus on the “finite-dimensional” version of (GE$(K,\infty )$) or (4.1), which we call *gradient estimate* $\mathrm {GE}(K,N)$.

#### Definition 4.1

Let $\Lambda$ be an operator mean and $(P_t)$ be a symmetric quantum Markov semigroup whose generator takes the Lindblad form (LB). We say that $(P_t)$ satisfies the gradient estimate $\mathrm {GE}(K,N)$ for $K\in \mathbb {R}, N\in (0,\infty ]$ if 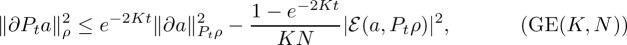 for any $t\ge 0$, $a\in \mathcal {M}$ and $\rho \in \mathcal {S}_+(\mathcal {M})$.

#### Remark 4.2

Both sides of (GE(*K*, *N*)) make sense for arbitrary $\rho \in {\mathcal {S}}(\mathcal {M})$ and are continuous in $\rho$. Thus, if $\rho \in \mathcal {S(M)}$ is not invertible, one can apply (GE(*K*, *N*)) to $\rho ^\epsilon =\frac{\rho +\epsilon {\mathbf {1}}}{1+\epsilon }$, which is invertible for $\epsilon >0$, and let $\epsilon \searrow 0$ to see that

$$\begin{aligned} \Vert \partial P_t a\Vert _\rho ^2\le e^{-2Kt}\Vert \partial a\Vert _{P_t\rho }^2-\frac{1-e^{-2Kt}}{KN} |\mathcal {E}( a,P_t\rho )|^2 \end{aligned}$$

still holds for any $t\ge 0$ and $a\in \mathcal {M}$.

#### Remark 4.3

It is obvious that when $N=\infty$, (GE(*K*, *N*)) becomes the gradient estimate $\mathrm {GE}(K,\infty )$. From the definition it is not immediately clear that if $(P_t)$ satisfies the gradient estimate $\mathrm {GE}(K,N)$, then it also satisfies the gradient estimate $\mathrm {GE}(K',N')$ whenever $K'\le K$ and $N'\ge N$. But this can be seen from the following equivalent formulation in the flavor of the $\Gamma _2$-condition.

#### Proposition 4.4

For any operator mean $\Lambda$ and any symmetric quantum Markov semigroup $(P_t)$, the gradient estimate (GE(*K*, *N*)) holds if and only if

4.2 $$\begin{aligned} {\text {Re}}\langle \partial \mathcal {L}a,{\hat{\rho }}\partial a\rangle -\frac{1}{2}\langle dG(\rho )(\mathcal {L}\rho )\partial a,\partial a\rangle \ge K \Vert \partial a\Vert _{\rho }^2+\frac{1}{N}|\mathcal {E}( a,\rho )|^2 \end{aligned}$$

for any $\rho \in \mathcal {S}_+(\mathcal {M})$ and any $a\in \mathcal {M}$. Here $dG(\rho )$ denotes the Fréchet derivative of $G(\rho ):={\hat{\rho }}=\Lambda (L(\rho ),R(\rho ))$.

#### Proof

Assume that $(P_t)$ satisfies (GE(*K*, *N*)). Set

$$\begin{aligned} \phi (t):=e^{-2Kt}\Vert \partial a\Vert _{P_t\rho }^2-\Vert \partial P_t a\Vert _\rho ^2-\frac{1-e^{-2Kt}}{KN}|\mathcal {E}(a,P_t \rho )|^2. \end{aligned}$$

Then $\phi (t)\ge 0$ and $\phi (0)=0$. Therefore $\phi '(0)\ge 0$, that is,

$$\begin{aligned} \left\langle \frac{\mathrm{d}}{\mathrm{d}t}\big |_{t=0}\widehat{P_t \rho }\partial a,\partial a\right\rangle -2K\Vert \partial a\Vert _{\rho }^2 +\langle {\hat{\rho }}\partial \mathcal {L}a,\partial a\rangle +\langle {\hat{\rho }}\partial a,\partial \mathcal {L}a\rangle -\frac{2}{N}|\mathcal {E}( a,\rho )|^2\ge 0. \end{aligned}$$

This is nothing but (4.2), since $dG(\rho )(\mathcal {L}\rho )=-\frac{\mathrm{d}}{\mathrm{d}t}\big |_{t=0}\widehat{P_t \rho }$.

Now suppose that $(P_t)$ satisfies 4.2. Fix $t> 0$ and put

$$\begin{aligned} \varphi (s):=e^{-2Ks}\Vert \partial P_{t-s} a\Vert _{P_{s}\rho }^2,~~0\le s\le t. \end{aligned}$$

Then applying (4.2) to $(\rho ,a)=(P_s\rho ,P_{t-s}a)$, we get

$$\begin{aligned} \varphi '(s)=\,&e^{-2Ks}\left( \langle \widehat{P_s\rho }\partial \mathcal {L}P_{t-s}a,\partial P_{t-s}a\rangle +\langle \widehat{P_s\rho }\partial P_{t-s} a,\partial \mathcal {L}P_{t-s} a\rangle \right. \\&\left. -\left\langle dG(P_s\rho )(\mathcal {L}\rho )\partial P_{t-s}a,\partial P_{t-s} a\right\rangle -2K\Vert \partial P_{t-s} a\Vert _{P_{s}\rho }^2\right) \\ \ge&\frac{2}{N}e^{-2Ks}|\mathcal {E}(P_{t-s} a,P_s\rho )|^2\\ =&\frac{2}{N}e^{-2Ks}|\mathcal {E}(a,P_{t}\rho )|^2. \end{aligned}$$

This, together with the fundamental theorem of calculus, yields

$$\begin{aligned} e^{-2Kt}\Vert \partial a\Vert _{P_t\rho }^2- \Vert \partial P_t a\Vert _\rho ^2 =\varphi (t)-\varphi (0) =\int _{0}^{t}\varphi '(s)\mathrm{d}s \ge \frac{1-e^{-2Kt}}{KN}|\mathcal {E}(a,P_t\rho )|^2. \end{aligned}$$

Therefore $(P_t)$ satisfies (GE(*K*, *N*)). $\square$

#### Remark 4.5

Similar to Remark 3.2, the function

$$\begin{aligned} t\mapsto \frac{1-e^{-2Kt}}{KN}, \end{aligned}$$

in (GE(*K*, *N*)) can be replaced by any *f* such that $f(0)=0$ and $f'(0)=2/N$.

#### Remark 4.6

In the case $N=\infty$, the gradient estimate $\mathrm {GE}(K,\infty )$ for the left trivial mean is equivalent to the exponential form of $\mathrm {BE}(K,\infty )$. For $N<\infty$ this seems to be no longer the case, but one still has one implication: the Bakry–Émery curvature-dimension condition BE(*K*, *N*) is stronger than $\mathrm {GE}(K,N)$ for the left trivial mean. This is a consequence of Cauchy–Schwarz inequality for the state $\tau (\rho \,\cdot \,)$:

$$\begin{aligned} |\mathcal {E}(a,\rho )|^2=|\langle \mathcal {L}a,\rho \rangle |^2\le \langle |\mathcal {L}a|^2,\rho \rangle . \end{aligned}$$

Similar to BE(*K*, *N*), the intertwining condition is also sufficient to prove $\mathrm {GE}(K,N)$ with the same dimension (upper bound).

#### Theorem 4.7

Let $(P_t)$ be a symmetric quantum Markov semigroup over $\mathcal {M}$ with the Lindblad form (LB). Suppose that $(P_t)$ satisfies *K*-intertwining condition for some $K\in \mathbb {R}$. Then for any operator mean $\Lambda$ the quantum Markov semigroup $(P_t)$ satisfies $\mathrm {GE}(K,d)$.

#### Proof

For $a\in \mathcal {M}$, recall that

$$\begin{aligned} P_t(a^*a)-(P_t a)^*P_t a =\int _{0}^{t}\frac{\mathrm{d}}{\mathrm{d}s} P_{s}\left( (P_{t-s} a)^*P_{t-s} a\right) ds =2\int _{0}^{t} P_{s}\Gamma (P_{t-s} a) \mathrm{d}s. \end{aligned}$$

Under the *K*-intertwining condition, we have (either by Kadison–Schwarz or BE($K,\infty$))

$$\begin{aligned} P_{s}\Gamma (P_{t-s} a)\ge e^{2Ks}\Gamma (P_t a). \end{aligned}$$

So

4.3 $$\begin{aligned} P_t(a^*a)-(P_t a)^*P_t a \ge 2\int _{0}^{t}e^{2Ks}\mathrm{d}s\Gamma (P_t a) =\frac{e^{2Kt}-1}{K}\Gamma (P_t a). \end{aligned}$$

By (3.2) and Lemma 3.5, we get for any $(x_j)_{1\le j\le d}\subset \mathcal {M}$

4.4 $$\begin{aligned} \sum _{j=1}^{d}\Gamma (P_t x_j)= & {} \sum _{j,k=1}^{d}|\partial _k P_t x_j|^2 =\sum _{j,k=1}^{d}|\partial _k^\dagger P_t x_j|^2 \ge \sum _{j=1}^{d}|\partial _j^\dagger P_t x_j|^2 \nonumber \\\ge & {} \frac{1}{d}\left| \sum _{j=1}^{d}\partial _j^\dagger P_t x_j\right| ^2. \end{aligned}$$

Let ${\hat{\mathcal {M}}}=\oplus _{j=1}^{d}\mathcal {M}$ be equipped with the inner product

$$\begin{aligned} \langle (x_j),(y_j)\rangle :=\sum _{j=1}^{d}\langle x_j,y_j\rangle , \end{aligned}$$

and ${\hat{P}}_t$ be the operator acting on ${\hat{\mathcal {M}}}$ such that ${\hat{P}}_t(x_1,\dots ,x_d)=(P_t x_1,\dots , P_t x_d)$. Fix $\rho \in \mathcal {S}_+(\mathcal {M})$. For simplicity, let us identify $\rho$ with the element $(\rho ,\dots ,\rho )$ in ${\hat{\mathcal {M}}}$. Then for $x=(x_1,\dots ,x_d)\in {\hat{\mathcal {M}}}$, we have by (4.3) and (4.4) that

$$\begin{aligned} \langle {\hat{P}}_t(x^*x),\rho \rangle -\langle ({\hat{P}}_t x)^*{\hat{P}}_t x,\rho \rangle =&\sum _{j=1}^{d}\langle P_t(x_j^*x_j)-(P_t x_j)^*P_t x_j,\rho \rangle \\ \ge&\frac{e^{2Kt}-1}{K}\sum _{j=1}^{d}\langle \Gamma (P_t x_j),\rho \rangle \\ \ge&\frac{e^{2Kt}-1}{dK}\left\langle \left| \sum _{j=1}^{d} \partial _j^\dagger P_t x_j\right| ^2,\rho \right\rangle . \end{aligned}$$

From *K*-intertwining condition and Cauchy–Schwarz inequality for the state $\tau (\rho \cdot )$ on $\mathcal {M}$, this is bounded from below by

$$\begin{aligned} \frac{1-e^{-2Kt}}{dK}\left\langle \left| \sum _{j=1}^{d} P_t\partial _j^\dagger x_j\right| ^2,\rho \right\rangle\ge & {} \frac{1-e^{-2Kt}}{dK}\left| \sum _{j=1}^{d}\left\langle P_t\partial _j^\dagger x_j,\rho \right\rangle \right| ^2\\= & {} \frac{1-e^{-2Kt}}{dK}\left| \left\langle x,\partial P_t \rho \right\rangle \right| ^2. \end{aligned}$$

So we have proved that for any $x\in {\hat{\mathcal {M}}}$:

$$\begin{aligned} \langle x({\hat{P}}_t \rho ),x\rangle \ge \langle {\hat{P}}_t x,({\hat{P}}_t x)\rho \rangle +\frac{1-e^{-2Kt}}{dK}\langle x,\partial P_t \rho \rangle \langle \partial P_t\rho ,x\rangle , \end{aligned}$$

or equivalently

$$\begin{aligned} R(P_t \rho )\ge {\hat{P}}_t R(\rho ){\hat{P}}_t +\frac{1-e^{-2Kt}}{dK}|\partial P_t \rho \rangle \langle \partial P_t\rho |. \end{aligned}$$

Replacing *x* by $x^*$, we obtain

$$\begin{aligned} L(P_t \rho )\ge {\hat{P}}_t L(\rho ){\hat{P}}_t+\frac{1-e^{-2Kt}}{dK}|\partial P_t \rho \rangle \langle \partial P_t\rho |. \end{aligned}$$

Note that the second summand is the same in both cases.

Now since $\Lambda$ is an operator mean, we have

$$\begin{aligned} \Lambda (L(P_t\rho ),R(P_t\rho )) \ge&\Lambda ({\hat{P}}_t L(\rho ){\hat{P}}_t,{\hat{P}}_t R(\rho ){\hat{P}}_t)\\&\quad +\frac{1-e^{-2Kt}}{dK}\Lambda \left( |\partial P_t \rho \rangle \langle \partial P_t\rho |,|\partial P_t \rho \rangle \langle \partial P_t\rho |\right) \\ \ge&{\hat{P}}_t \Lambda (L(\rho ),R(\rho )){\hat{P}}_t+\frac{1-e^{-2Kt}}{dK}|\partial P_t \rho \rangle \langle \partial P_t\rho |, \end{aligned}$$

where in the first inequality we used the monotonicity, concavity (Lemma 2.1 (b)) and positive homogeneity (Lemma 2.1 (a)) of $\Lambda$, and in the second inequality we used the transformer inequality and Lemma 2.1(d). This, together with the *K*-intertwining condition, yields

$$\begin{aligned} \Vert \partial P_t a\Vert _\rho ^2&=\langle \Lambda (L(\rho ),R(\rho ))\partial P_t a,\partial P_t a\rangle \\&= e^{-2Kt}\langle {\hat{P}}_t \Lambda (L(\rho ),R(\rho )){\hat{P}}_t \partial a,\partial a\rangle \\&\le e^{-2Kt}\langle \Lambda (L(P_t\rho ),R(P_t\rho ))\partial a,\partial a\rangle - \frac{e^{-2Kt}-e^{-4Kt}}{dK}|\langle \partial P_t \rho ,\partial a\rangle |^2\\&=e^{-2Kt}\Vert \partial a\Vert _{P_t\rho }^2-\frac{e^{-2Kt}-e^{-4Kt}}{dK} |\mathcal {E}(a,P_t\rho )|^2. \end{aligned}$$

This completes the proof, by Remark 4.5. $\square$

### Bonnet–Myers Theorem

As a first application of the dimensional gradient estimate $\mathrm {GE}(K,N)$, we present here a Bonnet–Myers theorem for the noncommutative analog of the Wasserstein distance introduced in [11, 12]. The proof is quite similar (or, in fact, similar to the dual) to the proof of Proposition 3.9.

Let us first recall the definition of the metric. The space $\mathcal {S}_+(\mathcal {M})$ of invertible density matrices is a smooth manifold and the tangent space at $\rho \in \mathcal {S}_+(\mathcal {M})$ can be canonically identified with the traceless self-adjoint elements of $\mathcal {M}$. Assume that $(P_t)$ is an *ergodic* tracially symmetric quantum Markov semigroup with generator $\mathcal {L}$ with Lindblad form (LB).

Fix an operator mean $\Lambda$. For $\rho \in {\mathcal {S}}_+(\mathcal {M})$ we define

4.5 $$\begin{aligned} \mathcal {K}_\rho ^\Lambda :\mathcal {M}\rightarrow \mathcal {M},\,x\mapsto \partial ^\dagger {\hat{\rho }}\partial x=\sum _{j=1}^d\partial _j^\dagger (\Lambda (L(\rho ),R(\rho ))\partial _j(x)). \end{aligned}$$

It maps $\{x\in \mathcal {M}\mid \tau (x)=0\}$ bijectively onto itself.

The Riemannian metric $g^\Lambda$ on $\mathcal {S}_+(\mathcal {M})$ is defined by

$$\begin{aligned} g^\Lambda _\rho ({\dot{\rho }}_1(0),{\dot{\rho }}_2(0))=\langle {\dot{\rho }}_1(0),(\mathcal {K}^\Lambda _\rho )^{-1}{\dot{\rho }}_2(0)\rangle , \end{aligned}$$

where $\rho _1$, $\rho _2$ are smooth curves in $\mathcal {S}_+(\mathcal {M})$ with $\rho _1(0)=\rho _2(0)=\rho$. In particular, $\tau ({\dot{\rho }}_j(0))=\frac{\mathrm{d}}{\mathrm{d}t}|_{t=0}\tau (\rho _j(t))=0$ for $j\in \{1,2\}$, so that the inverse of $\mathcal {K}_\rho ^\Lambda$ is well-defined.

The associated distance function on $\mathcal {S}_+(\mathcal {M})\times \mathcal {S}_+(\mathcal {M})$ is denoted by $\mathcal {W}$. By [12, Proposition 9.2], $\mathcal {W}$ can be extended to $\mathcal {S}(\mathcal {M})\times \mathcal {S}(\mathcal {M})$ since

$$\begin{aligned} \Lambda (a{\mathbf {1}},b{\mathbf {1}})\ge \Lambda (\min \{a,b\}{\mathbf {1}},\min \{a,b\}{\mathbf {1}})=\min \{a,b\}{\mathbf {1}}\end{aligned}$$

for all $$a,b>0$$.

#### Proposition 4.8

Fix an operator mean $\Lambda$. Let $K,N\in (0,\infty )$. If $(P_t)$ satisfies the gradient estimate $\mathrm {GE}(K,N)$, then

$$\begin{aligned} \mathcal {W}(\rho ,{\mathbf {1}})\le \frac{\pi }{2}\sqrt{\frac{N}{K}} \end{aligned}$$

for all $$\rho \in \mathcal {S}_+(\mathcal {M})$$.

In particular,

$$\begin{aligned} \sup _{\rho _0,\rho _1\in \mathcal {S}_+(\mathcal {M})}\mathcal {W}(\rho _0,\rho _1)\le \pi \sqrt{\frac{N}{K}}. \end{aligned}$$

#### Proof

Since $(P_t)$ is ergodic, we have $P_t \rho \rightarrow {\mathbf {1}}$ as $t\rightarrow \infty$. Let $\rho _t=P_t \rho$ for $t\ge 0$. The gradient estimate $\mathrm {GE}(K,N)$ implies

$$\begin{aligned} |\langle a,{\dot{\rho }}_t\rangle |=|\langle a,\mathcal {L}\rho _t\rangle |\le \sqrt{KN}\sqrt{\frac{e^{-2Kt}}{1-e^{-2Kt}}}\Vert \partial a\Vert _{\rho _t}=\frac{\sqrt{KN}}{\sqrt{e^{2Kt}-1}}\Vert \partial a\Vert _{\rho _t} \end{aligned}$$

for all $a\in \mathcal {M}$. Choosing $a=(\mathcal {K}^\Lambda _{\rho _t})^{-1}{\dot{\rho }}_t$, we get

$$\begin{aligned} g^{\Lambda }_{\rho _t}({\dot{\rho }}_t,{\dot{\rho }}_t) \le \frac{\sqrt{KN}}{\sqrt{e^{2Kt}-1}}\sqrt{\langle \mathcal {K}^\Lambda _{\rho _t} a,a\rangle } = \frac{\sqrt{KN}}{\sqrt{e^{2Kt}-1}}\sqrt{g^{\Lambda }_{\rho _t}({\dot{\rho }}_t,\dot{\rho }_t)}. \end{aligned}$$

Hence

$$\begin{aligned} \sqrt{g^{\Lambda }_{\rho _t}({\dot{\rho }}_t,{\dot{\rho }}_t)}\le \frac{\sqrt{KN}}{\sqrt{e^{2Kt}-1}}, \end{aligned}$$

and we conclude

$$\begin{aligned} \mathcal {W}(\rho ,{\mathbf {1}})\le \int _0^\infty \sqrt{g^{\Lambda }_{\rho _t}({\dot{\rho }}_t,\dot{\rho }_t)}\,\mathrm{d}t\le \int _0^\infty \frac{\sqrt{KN}}{\sqrt{e^{2Kt}-1}}\,\mathrm{d}t=\frac{\pi }{2} \sqrt{\frac{N}{K}}.\square \end{aligned}$$

#### Remark 4.9

This result can be extended to the non-ergodic case just as in Remark 3.10.

### Complete $\mathrm {GE}(K,N)$

Now we turn to the complete version of $\mathrm {GE}(K,N)$. In this part we always fix an operator mean $\Lambda$.

#### Definition 4.10

We say that a quantum Markov semigroup $(P_t)$ satisfies complete gradient estimate $\mathrm {CGE}(K,N)$ for $K\in \mathbb {R}$ and $N\in (0,\infty ]$ if $(P_t\otimes \mathrm {id}_{M_n(\mathbb {C})})$ satisfies $\mathrm {GE}(K,N)$ for all $n\in \mathbb {N}$ (for the fixed operator mean $\Lambda$).

Similar to Proposition 3.14, the *K*-intertwining condition is sufficient for $\mathrm {CGE}:$

#### Proposition 4.11

Suppose that the generator $\mathcal {L}$ of the quantum Markov semigroup $(P_t)$ admits the Lindblad form (LB) with *d* partial derivatives $\partial _1,\dots ,\partial _d$. If $(P_t)$ satisfies the *K*-intertwining condition for $K\in \mathbb {R}$, then $(P_t)$ satisfies $\mathrm {CGE}(K,d)$.

Also, the complete gradient estimate $\mathrm {CGE}$ is tensor stable.

#### Proposition 4.12

Consider two quantum Markov semigroups $(P_t^j)$ acting on $\mathcal {M}_j$, $j=1,2$. If $(P_t^j)$ satisfies $\mathrm {CGE}(K_j,N_j),j=1,2$, then the tensor product $(P_t^1\otimes P_t^2)$ over $\mathcal {M}=\mathcal {M}_1\otimes \mathcal {M}_2$ satisfies $\mathrm {CGE}(K,N)$ with $K=\min \{K_1,K_2\}$ and $N=N_1+ N_2$.

#### Proof

For each $j=1,2$, we denote by $\mathcal {L}_j$ the generator of $(P_t^j)$ and $\partial ^j:\mathcal {M}_j\rightarrow {\hat{\mathcal {M}}}_j$ (to distinguish from partial derivatives $\partial _j$’s) the corresponding derivation operator so that $\mathcal {L}_j=(\partial ^{j})^\dagger \partial ^j$. Denote $P_t=P_t^1\otimes P_t^2$. Then its generator is $\mathcal {L}=\partial ^\dagger \partial$, where the derivation operator $\partial$ is given by

$$\begin{aligned} \partial =(\partial ^1\otimes \mathrm {id},\mathrm {id}\otimes \partial ^2). \end{aligned}$$

Since $(P_t^j)$ satisfies $\mathrm {CGE}(K,N_j),j=1,2$, we have for any $a\in {\hat{\mathcal {M}}}:=\otimes _j{\hat{\mathcal {M}}}_j$ and $\rho \in \mathcal {S}_+(\mathcal {M})$ that

$$\begin{aligned} \Vert \partial P_t a\Vert _\rho ^2 =&\Vert (\partial ^1 \otimes \mathrm {id})(P_t^1\otimes \mathrm {id})(\mathrm {id}\otimes P_t^2)a\Vert _{\rho }^2+\Vert (\mathrm {id}\otimes \partial ^2)(\mathrm {id}\otimes P_t^2)(P_t^1 \otimes \mathrm {id})a\Vert _{\rho }^2\\ \le&e^{-2Kt}\left( \Vert (\partial ^1 \otimes \mathrm {id})(\mathrm {id}\otimes P_t^2)a\Vert _{(P_t^1\otimes \mathrm {id})\rho }^2 +\Vert (\mathrm {id}\otimes \partial ^2)(P_t^1 \otimes \mathrm {id})a\Vert _{(\mathrm {id}\otimes P_t^2)\rho }^2\right) \\&-\frac{1-e^{-2Kt}}{K}\left( \frac{1}{N_1}|\langle (\mathcal {L}_1\otimes \mathrm {id}) P_t a,\rho \rangle |^2+\frac{1}{N_2}|\langle (\mathrm {id}\otimes \mathcal {L}_2) P_t a,\rho \rangle |^2\right) . \end{aligned}$$

As we have proven in [39, Theorem 4.1], for the first summand one has

$$\begin{aligned} \Vert (\partial ^1 \otimes \mathrm {id})(\mathrm {id}\otimes P_t^2)a\Vert _{(P_t^1\otimes \mathrm {id})\rho }^2 +\Vert (\mathrm {id}\otimes \partial ^2)(P_t^1 \otimes \mathrm {id})a\Vert _{(\mathrm {id}\otimes P_t^2)\rho }^2\le \Vert \partial a\Vert _{P_t \rho }^2. \end{aligned}$$

As for the second summand, note that $\mathcal {L}=\mathcal {L}_1\otimes \mathrm {id}+\mathrm {id}\otimes \mathcal {L}_2$. So by Cauchy–Schwarz inequality,

$$\begin{aligned} \frac{1}{N} |\langle \mathcal {L}P_t a,\rho \rangle |^2&=\frac{|\langle (\mathcal {L}_1\otimes \mathrm {id}) P_t a,\rho \rangle +\langle (\mathrm {id}\otimes \mathcal {L}_2) P_t a,\rho \rangle |^2}{N_1+N_2}\\&\le \frac{1}{N_1}|\langle (\mathcal {L}_1\otimes \mathrm {id}) P_t a,\rho \rangle |^2+\frac{1}{N_2}|\langle (\mathrm {id}\otimes \mathcal {L}_2) P_t a,\rho \rangle |^2. \end{aligned}$$

All combined, we obtain

$$\begin{aligned} \Vert \partial P_t a\Vert _\rho ^2\le e^{-2Kt}\Vert \partial a\Vert _{P_t \rho }^2-\frac{1-e^{-2Kt}}{K N} |\langle \mathcal {L}P_t a,\rho \rangle |^2.\square \end{aligned}$$

## Geodesic (*K*, *N*)-Convexity of the (Relative) Entropy and Relation to the Gradient Estimate $\mathrm {GE}(K,N)$

In the case of the logarithmic mean, the given quantum Markov semigroup is the gradient flow of the (relative) entropy with respect to the transport distance $\mathcal {W}$. In this case, the gradient estimate $\mathrm {GE}(K,\infty )$ is equivalent to geodesic *K*-convexity of the (relative) entropy with respect to $\mathcal {W}$, and several functional inequalities can be obtained using gradient flow techniques.

Similarly, the gradient estimate $\mathrm {GE}(K,N)$ is equivalent to geodesic (*K*, *N*)-convexity of the (relative) entropy with respect to $\mathcal {W}$, a notion introduced by Erbar, Kuwada and Sturm [16], and again, gradient flow techniques allow to deduce several dimensional functional inequalities from the abstract theory of (*K*, *N*)-convex functions on Riemannian manifolds.

### (*K*, *N*)-Convexity for the (Relative) Entropy

Let (*M*, *g*) be a Riemannian manifold and $K\in \mathbb {R}$, $N\in (0,\infty ]$. A function $S\in C^2(M)$ is called (*K*, *N*)-*convex* if

$$\begin{aligned} {\text {Hess}}S(x)[v,v]-\frac{1}{N} g(\nabla S(x),v)^2\ge K g(v,v) \end{aligned}$$

for all $x\in M$ and $v\in T_x M$.

With the function

$$\begin{aligned} U_N:M\rightarrow \mathbb {R},\,U_N(x)=\exp \left( -\frac{1}{N} S(x)\right) , \end{aligned}$$

the (*K*, *N*)-convexity of *S* can equivalently be characterized by

$$\begin{aligned} {\text {Hess}}U_N\le -\frac{K}{N} U_N. \end{aligned}$$

For $N=\infty$, one obtains the usual notion of *K*-convexity. Moreover, the notion of (*K*, *N*)-convexity is obviously monotone in the parameters *K* and *N* in the sense that if *S* is (*K*, *N*)-convex, then *S* is also $(K',N')$-convex for $K'\le K$ and $N'\ge N$.

Our focus will be on the case when *F* is the (relative) entropy and the Riemannian metric is the one introduced in [11, 12], whose definition was recalled in Sect. 4.2.

If $F:\mathcal {S}_+(\mathcal {M})\rightarrow \mathbb {R}$ is smooth, its Fréchet derivative can be written as

$$\begin{aligned} d F(\rho )=\tau (x\,\cdot ) \end{aligned}$$

for a unique traceless self-adjoint $x\in \mathcal {M}$. This element *x* shall be denoted by $DF(\rho )$. In particular, if $F(\rho )=\tau (\rho \log \rho )$, then $DF(\rho )=\log \rho +c$ for some $c\in \mathbb {R}$.

By [11, Theorem 7.5], the gradient of *F* is given by (recall (4.5) for $\mathcal {K}_\rho ^\Lambda$)

5.1 $$\begin{aligned} \nabla _{g^\Lambda } F(\rho )=\mathcal {K}_\rho ^\Lambda DF(\rho ). \end{aligned}$$

Of particular interest to us is the case when *F* is the (relative) entropy, that is, the functional

$$\begin{aligned} {\text {Ent}}:\mathcal {S}_+(\mathcal {M})\rightarrow (0,\infty ),\,{\text {Ent}}(\rho )=\tau (\rho \log \rho ). \end{aligned}$$

If we choose $\Lambda$ to be the logarithmic mean $\Lambda _{\log }$, then $\rho _t=P_t \rho$ satisfies the gradient flow equation

$$\begin{aligned} {\dot{\rho }}_t=-\nabla _{g^\Lambda }{\text {Ent}}(\rho _t) \end{aligned}$$

for any $\rho \in \mathcal {S}_+(\mathcal {M})$ [11, Theorem 7.6]. For this reason, we fix the operator mean $\Lambda$ to be the logarithmic mean in this section.

To formulate the metric formulations of (*K*, *N*)-convexity, we need the following notation: For $\theta ,\kappa \in \mathbb {R}$ and $t\in [0,1]$ put

$$\begin{aligned} \begin{aligned} c_{\kappa }(\theta )&={\left\{ \begin{array}{ll}\cos (\sqrt{\kappa }\theta ),&{}\text {if }\kappa \ge 0,\\ \cosh (\sqrt{-\kappa }\theta ),&{}\text {if }\kappa<0,\end{array}\right. }\\ s_{\kappa }(\theta )&={\left\{ \begin{array}{ll}\kappa ^{-1/2}\sin (\sqrt{\kappa }\theta ),&{}\text {if }\kappa >0,\\ \theta ,&{}\text {if }\kappa =0,\\ (-\kappa )^{-1/2}\sinh (\sqrt{\kappa }\theta ),&{}\text {if }\kappa<0,\end{array}\right. }\\ \sigma _{\kappa }^{(t)}(\theta )&= {\left\{ \begin{array}{ll} \frac{s_{\kappa }(t\theta )}{s_{\kappa }(\theta )}, &{} \kappa \theta ^2\ne 0 \text { and }\kappa \theta ^2<\pi ^2,\\ t,&{}\kappa \theta ^2=0,\\ +\infty ,&{}\kappa \theta ^2\ge \pi ^2. \end{array}\right. } \end{aligned} \end{aligned}$$

The following theorem is a quite direct consequence of the abstract theory of (*K*, *N*)-convex functions and the computation of the gradient and Hessian on $(\mathcal {S}_+(\mathcal {M}),g)$ carried out in [11, 12]. Nonetheless, it implies some interesting functional inequalities, as we shall see in the following subsection.

#### Theorem 5.1

Fix the logarithmic mean $\Lambda =\Lambda _{\log }$. Let $K\in \mathbb {R}$ and $N\in (0,\infty ]$. Further let

$$\begin{aligned} U_N(\rho )=\exp \left( -\frac{1}{N} {\text {Ent}}(\rho )\right) . \end{aligned}$$

The following assertions are equivalent:

- The (relative) entropy ${\text {Ent}}$ is (*K*, *N*)-convex on $(\mathcal {S}_+(\mathcal {M}), g^{\Lambda })$.
- For all $\rho ,\nu \in \mathcal {S}_+(\mathcal {M})$, the following Evolution Variational Inequality holds for all $t\ge 0$: 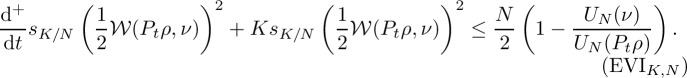
- For any constant speed geodesic $(\rho _t)_{t\in [0,1]}$ in $\mathcal {S}_+(\mathcal {M})$ one has
  $$\begin{aligned} U_N(\rho _t)\ge \sigma ^{(1-t)}_{K/N}(\mathcal {W}(\rho _0,\rho _1))U_N(\rho _0)+\sigma ^{(t)}_{K/N}(\mathcal {W}(\rho _0,\rho _1))U_N(\rho _1),~~t\in [0,1]. \end{aligned}$$
- The semigroup $(P_t)$ satisfies $\mathrm {GE}(K,N)$.

#### Proof

(a) $\iff$ (b)$\iff$(c): These equivalences follow from abstract theory of (*K*, *N*)-convex functionals on Riemannian manifolds [16, Lemmas 2.2, 2.4].

(a)$\iff$(d): With the identification of the gradient from (5.1) and the Hessian from [12, Proposition 7.16], one sees that the defining inequality of the (*K*, *N*)-convexity of *D* coincides with the equivalent formulation of $\mathrm {GE}(K,N)$ given in Proposition 4.4. $\square$

### Dimension-Dependent Functional Inequalities

Let us first collect some consequences of (*K*, *N*)-convexity that were already observed in [16], adapted to our setting. Recall that ${\text {Ent}}(\rho )=\tau (\rho \log \rho )$. We use the notation

$$\begin{aligned} \mathcal {I}(\rho )=\tau ((\mathcal {L}\rho )\log \rho ) \end{aligned}$$

for the Fisher information.

It satisfies the de Bruijn identity

$$\begin{aligned} \frac{\mathrm{d}}{\mathrm{d}t}{\text {Ent}}(P_t \rho )=-\mathcal {I}(P_t \rho ). \end{aligned}$$

The following inequalities (b) (c) and (d) are finite-dimensional versions of the HWI-inequality, modified log-Sobolev inequality (MLSI) and Talagrand inequality, respectively. The infinite-dimensional results (i.e. $N=\infty$) were obtained in [11, 12, 15].

#### Proposition 5.2

Fix the logarithmic mean $\Lambda =\Lambda _{\log }$. Let $K\in \mathbb {R}$ and $N>0$. If $(P_t)$ satisfies $\mathrm {GE}(K,N)$, then the following functional inequalities hold:

- $$\mathcal {W}$$-expansion bound:
  $$\begin{aligned}&s_{K/N}\left( \frac{1}{2}\mathcal {W}(P_t\rho _0,P_s\rho _1)\right) ^2\\&\quad \le e^{-K(s+t)}s_{K/N}\left( \frac{1}{2}\mathcal {W}(\rho _0,\rho _1)\right) ^2+\frac{N}{K}\left( 1-e^{-K(s+t)}\right) \frac{(\sqrt{t}-\sqrt{s})^2}{2(s+t)} \end{aligned}$$
  for $$\rho _0,\rho _1\in \mathcal {S}_+(\mathcal {M})$$ and $$s,t\ge 0$$.
- *N*-HWI inequality:
  $$\begin{aligned} \frac{U_N(\rho _1)}{U_N(\rho _0)}\le c_{K/N}(\mathcal {W}(\rho _0,\rho _1))+\frac{1}{N} s_{K/N}(\mathcal {W}(\rho _0,\rho _1))\sqrt{\mathcal {I}(\rho _0)}, \end{aligned}$$
  for $$\rho _0,\rho _1\in \mathcal {S}_+(\mathcal {M})$$ and $$s,t\ge 0$$.

If $K>0$, then additionally the following functional inequalities hold:

(c) *N*-MLSI:
  $$\begin{aligned} KN\left( U_N(\rho )^{-2}-1\right) \le \mathcal {I}(\rho ), \end{aligned}$$
  for $$\rho \in \mathcal {S}_+(\mathcal {M})$$.
(d) *N*-Talagrand inequality:
  $$\begin{aligned} {\text {Ent}}(\rho )\ge -N\log \cos \left( \sqrt{\frac{K}{N}}\mathcal {W}(\rho ,{\mathbf {1}})\right) , \end{aligned}$$
  for $$\rho \in \mathcal {S}_+(\mathcal {M})$$.

#### Proof

The proofs of Theorems 2.19, 3.28 and Corollaries 3.29, 3.31 from [16] can be easily adapted to our setting. $\square$

### Concavity of Entropy Power

Let us now move on to the concavity of entropy power:

$$\begin{aligned} t\mapsto U_N(P_t\rho )^2=\exp \left( -\frac{2}{N}{\text {Ent}}(P_t\rho )\right) . \end{aligned}$$

For the heat semigroup on $\mathbb {R}^n$, the concavity of entropy power along the heat flow was first proved by Costa in [13]. In [36] Villani gave a short proof and remarked that this can be proved using $\Gamma _2$-calculus. Recently Li and Li [27] considered this problem on the Riemannian manifold under the curvature-dimension condition CD(*K*, *N*). Here we show that the geodesic concavity of the entropy power follows from the (*K*, *N*)-convexity of the entropy.

#### Theorem 5.3

Let $K\in \mathbb {R}$ and $N>0$. If $(P_t)$ satisfies $\mathrm {GE}(K,N)$ for the logarithmic mean, then

$$\begin{aligned} \frac{\mathrm{d}^2}{\mathrm{d}t^2}U_N(P_t\rho )^2\le -2K \frac{\mathrm{d}}{\mathrm{d}t} U_N(P_t \rho )^2,~~t\ge 0. \end{aligned}$$

In particular, if $K\ge 0$, then $\frac{\mathrm{d}^2}{\mathrm{d}t^2}U_N(P_t \rho )^2\le 0$. This implies the concavity of the entropy power $t\mapsto U_N(P_t \rho )^2$.

#### Proof

Let $\rho _t=P_t \rho$. Since ${\text {Ent}}$ is (*K*, *N*)-convex by Theorem 5.1 and $(P_t)$ is a gradient flow of ${\text {Ent}}$ in $(\mathcal {S}_+(\mathcal {M}),g)$ by our choice of the operator mean, we have

$$\begin{aligned} \frac{\mathrm{d}^2}{\mathrm{d}t^2}U_N(\rho _t)^2&=\frac{\mathrm{d}}{\mathrm{d}t}\left( -\frac{2}{N} \langle \nabla _g {\text {Ent}}(\rho _t),{\dot{\rho }}_t\rangle U_N(\rho _t)\right) \\&=\frac{\mathrm{d}}{\mathrm{d}t}\left( \frac{2}{N}\langle {\dot{\rho }}_t,{\dot{\rho }}_t\rangle U_N(\rho _t)^2\right) \\&=\left( \frac{4}{N} \langle {\dot{\rho }}_t,\nabla _{{\dot{\rho }}_t}{\dot{\rho }}_t\rangle +\frac{4}{N^2} \langle {\dot{\rho }}_t,{\dot{\rho }}_t\rangle ^2\right) U_N(\rho _t)^2\\&=\frac{4}{N}\left( -{\text {Hess}}{\text {Ent}}(\rho _t)[{\dot{\rho }}_t,{\dot{\rho }}_t]+\frac{1}{N} \langle \nabla {\text {Ent}}(\rho _t),{\dot{\rho }}_t\rangle ^2\right) U_N(\rho _t)^2\\&\le -\frac{4K}{N}\langle {\dot{\rho }}_t,{\dot{\rho }}_t\rangle U_N(\rho _t)^2\\&=-2K\frac{\mathrm{d}}{\mathrm{d}t}U_N(\rho _t)^2.\square \end{aligned}$$

#### Remark 5.4

The same proof applies abstractly whenever *F* is a (*K*, *N*)-convex functional on a Riemannian manifold and $(\rho _t)$ is a gradient flow curve of *F*.

The following proof is closer to the spirit of Villani.

#### Another proof of Theorem 5.3

Put $\varphi (t):=-{\text {Ent}}(\rho _t)=-\tau (\rho _t\log \rho _t)$ with $\rho _t=P_t\rho$. Recall the chain rule

$$\begin{aligned} \partial \rho ={\hat{\rho }}\partial \log \rho . \end{aligned}$$

Thus

5.2 $$\begin{aligned} \varphi '(t) =\langle \mathcal {L}\rho _t,\log \rho _t\rangle =\langle \widehat{\rho _t}\partial \log \rho _t),\partial \log \rho _t\rangle . \end{aligned}$$

This allows to give two forms of $\varphi ''$:

5.3 $$\begin{aligned} \varphi ''(t)=\frac{\mathrm{d}}{\mathrm{d}t}\langle \mathcal {L}\rho _t, \log \rho _t\rangle =\langle \mathcal {L}\rho _t, \frac{\mathrm{d}}{\mathrm{d}t} \log \rho _t\rangle -\langle \mathcal {L}\rho _t,\mathcal {L}\log \rho _t\rangle =:\mathrm {I}, \end{aligned}$$

and

5.4 $$\begin{aligned} \varphi ''(t)&=\frac{\mathrm{d}}{\mathrm{d}t}\langle \widehat{\rho _{t}}\partial \log \rho _t,\partial \log \rho _t\rangle \nonumber \\&=2 \langle \widehat{\rho _t}\partial \log \rho _t,\partial \frac{\mathrm{d}}{\mathrm{d}r}\big |_{r=t}\log \rho _r\rangle +\langle \frac{\mathrm{d}}{\mathrm{d}r}\big |_{r=t}\widehat{\rho _{r}}\partial \log \rho _t,\partial \log \rho _t\rangle \nonumber \\&=2\langle \mathcal {L}\rho _t, \frac{\mathrm{d}}{\mathrm{d}t} \log \rho _t\rangle +\langle \frac{\mathrm{d}}{\mathrm{d}r}\big |_{r=t}\widehat{\rho _{r}}\partial \log \rho _t,\partial \log \rho _t\rangle =:\mathrm {II}. \end{aligned}$$

From (5.3) and (5.4) we deduce that

5.5 $$\begin{aligned} \varphi ''(t)=2\mathrm {I}-\mathrm {II} =-2\langle \mathcal {L}\rho _t,\mathcal {L}\log \rho _t\rangle -\langle \frac{\mathrm{d}}{\mathrm{d}r}\big |_{r=t}\widehat{\rho _{r}}\partial \log \rho _t,\partial \log \rho _t\rangle . \end{aligned}$$

Since $(P_t)$ satisfies $\mathrm {GE}(K,N)$ we have by Proposition 4.4 that

$$\begin{aligned}&\langle \widehat{\rho _t}\partial \mathcal {L}\log \rho _t,\partial \log \rho _t\rangle +\frac{1}{2}\langle \frac{\mathrm{d}}{\mathrm{d}r}\big |_{r=t}\widehat{\rho _r}\partial \log \rho _t,\partial \log \rho _t\rangle \ge K \Vert \partial \log \rho _t\Vert _{\rho _t}^2\\&\quad +\frac{1}{N}|\mathcal {E}( \log \rho _t,\rho _t)|^2, \end{aligned}$$

that is,

$$\begin{aligned}&2\langle \mathcal {L}\log \rho _t,\mathcal {L}\rho _t\rangle +\langle \frac{\mathrm{d}}{\mathrm{d}r}\big |_{r=t}\widehat{\rho _r}\partial \log \rho _t,\partial \log \rho _t\rangle \\&\quad \ge 2K \Vert \partial \log \rho _t\Vert _{\rho _t}^2+\frac{2}{N}|\mathcal {E}( \log \rho _t,\rho _t)|^2. \end{aligned}$$

This, together with (5.2) and (5.5), yields

5.6 $$\begin{aligned} \varphi ''(t) \le -2K\Vert \partial \log \rho _t\Vert _{\rho _t}^2-\frac{2}{N}|\mathcal {E}(\log \rho _t,\rho _t)|^2 =-2K\varphi '(t)-\frac{2}{N}\varphi '(t)^2.\nonumber \\ \end{aligned}$$

A direct computation gives

$$\begin{aligned} \frac{\mathrm{d}}{\mathrm{d}t}U_N(P_t\rho )^2=\frac{2}{N}U_N(P_t\rho )^2\varphi '(t), \end{aligned}$$

and

$$\begin{aligned} \frac{\mathrm{d}^2}{\mathrm{d}t^2}U_N(P_t\rho )^2=\frac{2}{N}U_N(P_t\rho )^2\left( \frac{2}{N}\varphi '(t)^2+\varphi ''(t)\right) . \end{aligned}$$

So by (5.6) we get

$$\begin{aligned} \frac{\mathrm{d}^2}{\mathrm{d}t^2}U_N(P_t\rho )^2 \le -\frac{4K}{N}U_N(P_t\rho )^2\varphi '(t) =-2K\frac{\mathrm{d}}{\mathrm{d}t}U_N(P_t\rho )^2.\square \end{aligned}$$

#### Remark 5.5

Here we used the fact that $\mathrm {I}=\mathrm {II},$ or equivalently,

$$\begin{aligned} \langle \mathcal {L}\rho _t, \frac{\mathrm{d}}{\mathrm{d}t}\log \rho _t\rangle +\langle \mathcal {L}\rho _t,\mathcal {L}\log \rho _t\rangle +\langle \frac{\mathrm{d}}{\mathrm{d}r}\big |_{r=t}\widehat{\rho _{r}}\partial \log \rho _t,\partial \log \rho _t\rangle =0. \end{aligned}$$

If we consider the heat semigroup $P_t=e^{t\Delta }$ on $\mathbb {R}^n$, then this follows from the elementary identity

$$\begin{aligned} \frac{\Delta f}{f}=\Delta (\log f)+|\nabla (\log f)|^2, \end{aligned}$$

as used in Villani’s proof [36].

## Examples

In this section we present several classes of examples of quantum Markov semigroups satisfying $\mathrm {CBE}(K,N)$ and $\mathrm {CGE}(K,N)$. The verification of these examples relies crucially on the criteria from Propositions 3.14 and 4.11.

### Schur Multipliers Over Matrix Algebras

A Schur multiplier *A* over the $n\times n$ matrix algebra $M_n(\mathbb {C})$ is a linear map of the form:

$$\begin{aligned} Ae_{ij}:=a_{ij}e_{ij}, \end{aligned}$$

where $a_{ij}\in \mathbb {C}$ and $\{e_{ij}\}_{i,j=1}^n$ are the matrix units. By Schoenberg’s theorem (see for example [9, Appendix D]),

$$\begin{aligned} P_t[x_{ij}]=e^{-tA} [x_{ij}]=[e^{-t a_{ij}}x_{ij}],~~t\ge 0, \end{aligned}$$

defines a symmetric quantum Markov semigroup over $M_n(\mathbb {C})$ if and only if

- $$a_{ii}=0$$ for all $$1\le i\le n$$,
- $$a_{ij}=a_{ji}\ge 0$$ for all $$1\le i,j\le n$$,
- $[a_{ij}]$ is *conditionally negative definite*:
  $$\begin{aligned} \sum _{i,j=1}^{n}\overline{\alpha _i}\alpha _ja_{ij}\le 0, \end{aligned}$$
  whenever $\alpha _1,\dots ,\alpha _n$ are complex numbers such that $\sum _{j=1}^{n}\alpha _j=0$.

If this is the case, then there exists a real Hilbert space *H* and elements $a(j)\in H$, $1\le j\le n$, such that

$$\begin{aligned} a_{ij}=\Vert a(i)-a(j)\Vert ^2,~~1\le i,j\le n. \end{aligned}$$

Suppose that $(e_k)_{1\le k\le d}$ is an orthonormal basis of *H*. Define for each $1\le k\le d$

$$\begin{aligned} v_k:=\sum _{j=1}^{n}\langle a(j),e_k\rangle e_{jj}\in M_n(\mathbb {C}). \end{aligned}$$

Then for any $1\le i,j\le n$:

$$\begin{aligned} {[}v_k,e_{ij}]=v_k e_{ij}- e_{ij}v_k=\langle a(i)-a(j),e_k\rangle e_{ij}, \end{aligned}$$

and

$$\begin{aligned} {[}v_k,[v_k,e_{ij}]]=|\langle a(i)-a(j),e_k\rangle |^2 e_{ij}. \end{aligned}$$

By the choice of $(e_k)$, we have

$$\begin{aligned} \sum _{k=1}^{d}[v_k,[v_k,e_{ij}]]=\Vert a(i)-a(j)\Vert ^2 e_{ij}=a_{ij}e_{ij}. \end{aligned}$$

Therefore,

$$\begin{aligned} A=\sum _{k=1}[v_k,[v_k,\cdot ]], \end{aligned}$$

and it is easy to see that $[v_k,A\cdot ]=A[v_k,\cdot ]$ for each *k*. So by Propositions 3.14 and 4.11 we have $\mathrm {CBE}(0,d)$ and $\mathrm {CGE}(0,d)$ for any operator mean.

### Herz–Schur Multipliers Over Group Algebras

Let *G* be a finite group. Suppose that $\lambda$ is the left-regular representation, i.e. for $g\in G$,where  is the delta function at *h*. The group algebra of *G* is then the (complex) linear span of $\{\lambda _g\mid g\in G\}$, denoted by $\mathbb {C}[G]$. It carries a canonical tracial state $\tau$ given by , where *e* is the unit element of *G*.

We say that $\ell :G\rightarrow [0,\infty )$ is a *conditionally negative definite length function* if $\ell (e)=0$, $\ell (g^{-1})=\ell (g)$ for all $g\in G$ and

$$\begin{aligned} \sum _{g,h\in G}\overline{\alpha _g} \alpha _h \ell (g^{-1}h)\le 0 \end{aligned}$$

whenever $\alpha _g$, $g\in G$, are complex numbers such that $\sum _{g\in G}\alpha _g=0$. By Schoenberg’s theorem (see for example [9, Appendix D]), there exists a 1-cocycle $(H,\pi , b)$ consisting of a real Hilbert space *H* of dimension $\dim H\le |G|-1$, a unitary representation $\pi :G\rightarrow B(H)$ and a map $b:G\rightarrow H$ satisfying the cocycle condition

$$\begin{aligned} b(gh)=b(g)+\pi (g)b(h) \end{aligned}$$

for $g,h\in G$ such that $\ell (g)=\Vert b(g)\Vert ^2$.

Every conditionally negative definite length function $\ell$ on *G* induces a $\tau$-symmetric quantum Markov semigroup $(P_t)$ on $\mathbb {C}[G]$ characterized by $P_t\lambda _g=e^{-t\ell (g)}\lambda _g$ for $g\in G$. Let $e_1,\dots ,e_d$ be an orthonormal basis of *H*. As argued in [39] (or similar to the Schur multipliers case), the generator $\mathcal {L}$ of $(P_t)$ can be written as

$$\begin{aligned} \mathcal {L}=\sum _{j=1}^d [v_j,[v_j,\cdot \,]] \end{aligned}$$

with $d=\dim H$ andThe operators $v_j$ are not contained in $\mathbb {C}[G]$ in general, but one can extend $\mathcal {L}$ to a linear operator on $B(\ell _2(G))$ by the same formula, and a direct computation shows $[v_j,\mathcal {L}\,\cdot \,]=\mathcal {L}[v_j,\cdot \,]$. By Propositions 3.14 and 4.11, $(P_t)$ satisfies $\mathrm {CBE}(0,d)$ and $\mathrm {CGE}(0,d)$ for any operator mean.

#### Example 6.1

The cyclic group $\mathbb {Z}_n=\{0,1,\dots ,n-1\}$; see [21, Example 5.9] or [39, Example 5.7]: Its group (von Neumann) algebra is spanned by $\lambda _k,0\le k\le n-1$. One can embed $\mathbb {Z}_n$ to $\mathbb {Z}_{2n}$, so let us assume that *n* is even. The word length of $k\in \mathbb {Z}_n$ is given by $\ell (k)=\min \{k,n-k\}$. The associated 1-cocycle can be chosen with $H=\mathbb {R}^{\frac{n}{2}}$ and $b:\mathbb {Z}_n\rightarrow \mathbb {R}^{\frac{n}{2}}$ via

$$\begin{aligned} b(k)={\left\{ \begin{array}{ll} 0,&{}k=0,\\ \sum _{j=1}^{k}e_j,&{}1\le k\le \frac{n}{2},\\ \sum _{j=k-\frac{n}{2}+1}^{\frac{n}{2}}e_j,&{}\frac{n}{2}+1\le k\le n-1, \end{array}\right. } \end{aligned}$$

where $(e_j)_{1\le j\le \frac{n}{2}}$ is an orthonormal basis of $\mathbb {R}^{\frac{n}{2}}$. Thus the quantum Markov semigroup associated with $\ell$ satisfies $\mathrm {CBE}(0,n/2)$ and $\mathrm {CGE}(0,n/2)$ for any operator mean.

#### Example 6.2

The symmetric group $S_n$; see [39, Example 5.8]: Let $\ell$ be the length function induced by the (non-normalized) Hamming metric, that is, $\ell (\sigma )=\#\{j : \sigma (j)\ne j\}$. Let $A_\sigma \in M_n(\mathbb {R})$ be the permutation matrix associated with $\sigma$, i.e., $A_\sigma \delta _j =\delta _{\sigma (j)}$. Then the associated cocycle is given by $H=L^2(M_n(\mathbb {R}),\frac{1}{2} \mathrm {tr})$, $b(\sigma )=A_\sigma -1$ and $\pi (\sigma )=A_\sigma$. Thus the quantum Markov semigroup associated with $\ell$ satisfies $\mathrm {CBE}(0,d)$ and $\mathrm {CGE}(0,d)$ for any operator mean with $d=\min \{|S_n|-1,n^2\}$.

### Generalized Depolarizing Semigroups

Let $\tau$ be the normalized trace on $M_d(\mathbb {C})$ and $E:M_d(\mathbb {C})\rightarrow M_d(\mathbb {C})$ a $\tau$-preserving conditional expectation. The Popa–Pimsner index of *E* [33] is defined as

$$\begin{aligned} C(E)=\inf \{c\ge 1\mid \rho \le c E(\rho )\text { for all }\rho \in {\mathcal {S}}(M_d(\mathbb {C}))\}. \end{aligned}$$

The completely bounded Pimsner–Popa index [18] is given by

$$\begin{aligned} C_{\mathrm {cb}}(E)=\sup _{m\in \mathbb {N}}C(E\otimes \mathrm {id}_{M_m(\mathbb {C})}). \end{aligned}$$

It is finite for any *E* and can be computed explicitly in terms of the multiplicities of $\mathrm {ran}(E)$ inside of $M_d({\mathbb {C}})$. In the special case when $E(a)=\tau (a){\mathbf {1}}$, we have $C(E)=d$ and $C_{\mathrm {cb}}(E)=d^2$.

The generalized depolarizing semigroup (or dephasing semigroup) associated with *E* is given by

$$\begin{aligned} P_t:M_d(\mathbb {C})\rightarrow M_d(\mathbb {C}),\,P_t(a)=e^{-t}a+(1-e^{-t})E(a). \end{aligned}$$

Let $\mathcal {L}=\mathrm {id}-E$ be the generator of $(P_t)$ with Lindblad form

$$\begin{aligned} \mathcal {L}=\sum _{j\in \mathcal {J}}\partial _j^\dagger \partial _j. \end{aligned}$$

Fix $k\in \mathcal {J}$. Since $\mathcal {L}E=0$, we have

$$\begin{aligned} \Vert \partial _k E(a)\Vert _2^2\le \sum _{j\in \mathcal {J}}\Vert \partial _j E(a)\Vert _2^2=\langle \mathcal {L}E(a),E(a)\rangle =0. \end{aligned}$$

Thus $\Vert \partial _k P_t a\Vert _\rho ^2=e^{-2t}\Vert \partial _k a\Vert _\rho ^2$. By positive homogeneity (Lemma 2.1 (a)) and concavity (Lemma 2.1 (b)) of the operator mean $\Lambda$, we get

$$\begin{aligned} \Vert \partial _k a\Vert _{P_t\rho }^2\ge e^{-t}\Vert \partial _k a\Vert _\rho ^2+(1-e^{-t})\Vert \partial _k a\Vert _{E(\rho )}^2. \end{aligned}$$

Note that by the Leibniz rule,

$$\begin{aligned} (\partial _k a)E(\rho )=\partial _k(a E(\rho ))-a\partial _k E(\rho )=\partial _k(a E(\rho )), \end{aligned}$$

that is, $R(E(\rho ))\partial _k=\partial _k R(E(\rho ))$. Similarly, we have $L(E(\rho ))\partial _k=\partial _k L(E(\rho ))$, so that functional calculus gives $\widehat{E(\rho )}\partial _k=\partial _k\widehat{E(\rho )}$. Moreover,

$$\begin{aligned} \mathcal {L}(a E(\rho ))=aE(\rho )-E(aE(\rho ))=(a-E(a))E(\rho )=(\mathcal {L}a)E(\rho ), \end{aligned}$$

that is, $\mathcal {L}R(E(\rho ))=R(E(\rho ))\mathcal {L}$. Again, this is also valid for $L(E(\rho ))$ instead of $R(E(\rho ))$ and implies $\mathcal {L}\widehat{E(\rho )}=\widehat{E(\rho )}\mathcal {L}$.

Hence

$$\begin{aligned} \Vert \partial a\Vert _{P_t\rho }^2&\ge e^{-t}\Vert \partial a\Vert _{\rho }^2+(1-e^{-t})\sum _{j\in \mathcal {J}}\langle \partial _j a,\partial _j(\widehat{E(\rho )}a)\rangle \\&=e^{-t}\Vert \partial a\Vert _\rho ^2+(1-e^{-t})\langle \mathcal {L}a,\widehat{E(\rho )}a\rangle \\&=e^{-t}\Vert \partial a\Vert _\rho ^2+(1-e^{-t})\langle \mathcal {L}a,\mathcal {L}(\widehat{E(\rho )}a)\rangle \\&=e^{-t}\Vert \partial a\Vert _\rho ^2+(1-e^{-t})\langle \mathcal {L}a,\widehat{E(\rho )}\mathcal {L}a\rangle , \end{aligned}$$

where we used $\mathcal {L}^2=\mathcal {L}$ in the second to last step.

By the definition of the Pimsner–Popa index,

$$\begin{aligned} P_t\rho =e^{-t}\rho +(1-e^{-t})E(\rho )\le (e^{-t}C(E)+(1-e^{-t}))E(\rho )\le C(E)E(\rho ). \end{aligned}$$

An application of Cauchy–Schwarz then yields

$$\begin{aligned} \langle x,xE(\rho )\rangle \ge \frac{1}{C(E)}\langle x,x P_t\rho \rangle \ge \frac{1}{C(E)} |\tau (x (P_t\rho ))|^2, \end{aligned}$$

that is, $R(E(\rho ))\ge \frac{1}{C(E)}|P_t\rho \rangle \langle P_t\rho |$. The same holds for $L(E(\rho ))$ instead of $R(E(\rho ))$, which together with the monotonicity of operator means implies

$$\begin{aligned} \langle x,\widehat{E(\rho )}x\rangle \ge \frac{1}{C(E)}|\tau (x (P_t\rho ))|^2. \end{aligned}$$

Therefore,

$$\begin{aligned} \Vert \partial a\Vert _{P_t\rho }^2&\ge e^{-t}\Vert \partial a\Vert _\rho ^2+(1-e^{-t})\langle \mathcal {L}a,\widehat{E(\rho )}\mathcal {L}a\rangle \\&\ge e^{-t}\Vert \partial a\Vert _\rho ^2+\frac{(1-e^{-t})}{C(E)}|(\mathcal {L}a)P_t\rho )|^2, \end{aligned}$$

or, equivalently,

$$\begin{aligned} \Vert \partial P_t a\Vert _\rho ^2\le e^{-t}\Vert \partial a\Vert _{P_t\rho }^2-f(t)\mathcal {E}(a,P_t \rho )^2 \end{aligned}$$

with $f(t)=\frac{e^{-t}-e^{-2t}}{C(E)}$. As $f(0)=0$ and $f'(0)=1/C(E)$, this means that $(P_t)$ satisfies $\mathrm {GE}(1/2,2C(E))$ for any operator mean.

Choosing $\Lambda$ as the right-trivial mean and not applying Cauchy–Schwarz, one obtains in a similar manner

$$\begin{aligned} \tau (\Gamma (P_t a)\rho )\le e^{-t}\tau (\Gamma (a)P_t \rho )-\frac{1-e^{-t}}{C(E)}\tau (|\mathcal {L}a|^2P_t \rho ). \end{aligned}$$

Differentiation at $t=0$ then gives $\mathrm {BE}(1/2,2 C(E))$ for $(P_t)$.

As the same argument can be applied to $(P_t\otimes \mathrm {id}_{M_m(\mathbb {C})})$, this shows that $(P_t)$ satisfies $\mathrm {CGE}(1/2,2C_{\mathrm {cb}}(E))$ for any operator mean and $\mathrm {CBE}(1/2,2C_{\mathrm {cb}}(E))$.

## Curvature-Dimension Conditions Without Assuming Tracial Symmetry

In plenty of applications one encounters quantum Markov semigroups that are not necessarily tracially symmetric, but only satisfy the detailed balance condition $\sigma$-DBC (with $\sigma \ne {\mathbf {1}}$) we mentioned in Sect. 2. Many of the results from this article still apply in this case, with one important caveat, as we will discuss here.

The definition of the Bakry–Émery gradient estimate $\mathrm {BE}(K,N)$ makes sense for arbitrary quantum Markov semigroups on matrix algebras without any change, and all the consequences we proved stay valid in this more general setting with essentially the same proofs.

The gradient estimate $\mathrm {GE}(K,N)$ relies on the Lindblad form of the generator of the semigroup. By Alicki’s theorem, a similar Lindblad form exists for generators of quantum Markov semigroups satisfying the $\sigma$-DBC, and the norms $\Vert \xi \Vert _\rho$ have been defined in this setting in [11, 12] – in fact, instead of a single operator mean one can choose a family of operator connections that depends on the index *j*. With this norm, one can formulate $\mathrm {GE}(K,N)$ as

$$\begin{aligned} \Vert \partial P_t a\Vert _\rho ^2\le e^{-2Kt}\Vert \partial a\Vert ^2_{P_t^\dagger \rho }-\frac{1-e^{-2Kt}}{KN}|\tau ((\mathcal {L}P_t a)\rho )|^2, \end{aligned}$$

where one now has to distinguish between $P_t$ and $P_t^\dagger$ because of the lack of tracial symmetry.

The connection between a generalization of the metric $\mathcal {W}$, the semigroup $(P_t)$ and the relative entropy still remains true in this more general setting with an appropriate modification of the definition of $\mathcal {W}$ [11, 12], so that the identification of $\mathrm {GE}(K,N)$ with the (*K*, *N*)-convexity condition for an entropy functional from Theorem 5.1 along with its applications also has an appropriate analog for quantum Markov semigroups satisfying the $\sigma$-DBC.

However, the criteria from Proposition 3.7 and Theorem 4.7, which actually allow us to verify $\mathrm {BE}(K,N)$ and $\mathrm {GE}(K,N)$ in concrete examples, rely crucially on the Lindblad form of generators of tracially symmetric quantum Markov semigroups and do not immediately carry over to the $\sigma$-detailed balance case. Thus the question of proving $\mathrm {BE}(K,N)$ and $\mathrm {GE}(K,N)$ for not necessarily tracially symmetric quantum Markov semigroups remains open, so its usefulness in this case is still to be proven.

## References

[1] Aza, N.J.B., Trujillo, D.A.B.: Entropy power inequality in fermionic quantum computation. arXiv e-prints, arXiv:2008.05532 (2020)

[2] Ambrosio L, Gigli N, Savaré G (2014). Calculus and heat flow in metric measure spaces and applications to spaces with Ricci bounds from below. Invent. Math..

[3] Ambrosio L, Gigli N, Savaré G (2014). Metric measure spaces with Riemannian Ricci curvature bounded from below. Duke Math. J..

[4] Ambrosio L, Gigli N, Savaré G (2015). Bakry–Émery curvature-dimension condition and Riemannian Ricci curvature bounds. Ann. Probab..

[5] Alicki R (1976). On the detailed balance condition for non-Hamiltonian systems. Rep. Math. Phys..

[6] Bakry, B., Émery, M.: Diffusions hypercontractives. In: Séminaire de probabilités, XIX, 1983/84, volume 1123 of Lecture Notes in Mathematics, pp. 177–206. Springer, Berlin (1985)

[7] Brannan, M., Gao, L., Junge, M.: Complete logarithmic Sobolev inequalities via Ricci curvature bounded below II. arXiv e-prints, arXiv:2008.12038 (2020)

[8] Brannan M, Gao L, Junge M (2022). Complete logarithmic Sobolev inequalities via Ricci curvature bounded below. Adv. Math..

[9] Brown NP, Ozawa N (2008). $$C^*$$-Algebras and Finite-Dimensional Approximations. Graduate Studies in Mathematics.

[10] Carlen EA, Maas J (2014). An analog of the 2-Wasserstein metric in non-commutative probability under which the fermionic Fokker–Planck equation is gradient flow for the entropy. Commun. Math. Phys..

[11] Carlen EA, Maas J (2017). Gradient flow and entropy inequalities for quantum Markov semigroups with detailed balance. J. Funct. Anal..

[12] Carlen EA, Maas J (2020). Non-commutative calculus, optimal transport and functional inequalities in dissipative quantum systems. J. Stat. Phys..

[13] Costa MHM (1985). A new entropy power inequality. IEEE Trans. Inf. Theory.

[14] De Palma G, Trevisan D (2018). The conditional entropy power inequality for bosonic quantum systems. Commun. Math. Phys..

[15] Datta, N., Rouzé, C.: Relating relative entropy, optimal transport and fisher information: a quantum HWI inequality. Ann. Henri Poincaré (2020)

[16] Erbar M, Kuwada K, Sturm K-T (2015). On the equivalence of the entropic curvature-dimension condition and Bochner’s inequality on metric measure spaces. Invent. Math..

[17] Erbar M, Maas J (2012). Ricci curvature of finite Markov chains via convexity of the entropy. Arch. Ration. Mech. Anal..

[18] Gao L, Junge M, LaRacuente N (2020). Relative entropy for von Neumann subalgebras. Int. J. Math..

[19] Huber S, König R, Vershynina A (2017). Geometric inequalities from phase space translations. J. Math. Phys..

[20] Junge M, Mei T (2010). Noncommutative Riesz transforms: a probabilistic approach. Am. J. Math..

[21] Junge M, Zeng Q (2015). Noncommutative martingale deviation and Poincaré type inequalities with applications. Probab. Theory Relat. Fields.

[22] Junge M, Zeng Q (2015). Subgaussian 1-cocycles on discrete groups. J. Lond. Math. Soc. (2).

[23] Kubo F, Ando T (1980). Means of positive linear operators. Math. Ann..

[24] König R, Smith G (2014). The entropy power inequality for quantum systems. IEEE Trans. Inf. Theory.

[25] Li, H.: Complete Sobolev type inequalities. arXiv e-prints, arXiv:2008.09278 (2020)

[26] Li, H., Junge, M., LaRacuente, N.: Graph Hörmander systems. arXiv e-prints, arXiv:2006.14578 (2020)

[27] Li, S., Li, X.-D.: On the Shannon entropy power on Riemannian manifolds and Ricci flow. arXiv e-prints, arXiv:2001.00410 (2020)

[28] Liu, S., Münch, F., Peyerimhoff, N.: Bakry–Émery curvature and diameter bounds on graphs. Calc. Var. Partial Differ. Equ. **57**(2):Paper No. 67, 9 (2018)

[29] Lott J, Villani C (2009). Ricci curvature for metric-measure spaces via optimal transport. Ann. Math. (2).

[30] Maas J (2011). Gradient flows of the entropy for finite Markov chains. J. Funct. Anal..

[31] Mielke A (2013). Geodesic convexity of the relative entropy in reversible Markov chains. Calc. Var. Partial Differ. Equ..

[32] Mittnenzweig M, Mielke A (2017). An entropic gradient structure for Lindblad equations and couplings of quantum systems to macroscopic models. J. Stat. Phys..

[33] Pimsner M, Popa S (1986). Entropy and index for subfactors. Ann. Sci. École Norm. Sup. (4).

[34] Sturm K-T (2006). On the geometry of metric measure spaces. I. Acta Math..

[35] Sturm K-T (2006). On the geometry of metric measure spaces. II. Acta Math..

[36] Villani C (2000). A short proof of the “concavity of entropy power”. IEEE Trans. Inf. Theory.

[37] von Renesse M-K, Sturm K-T (2005). Transport inequalities, gradient estimates, entropy, and Ricci curvature. Commun. Pure Appl. Math..

[38] Wirth, M.: A noncommutative transport metric and symmetric quantum Markov semigroups as gradient flows of the entropy. arXiv e-prints, arXiv:1808.05419 (2018)

[39] Wirth M, Zhang H (2021). Complete gradient estimates of quantum Markov semigroups. Commun. Math. Phys..

